# Patching-based deep-learning model for the inpainting of Bragg coherent diffraction patterns affected by detector gaps

**DOI:** 10.1107/S1600576724004163

**Published:** 2024-06-18

**Authors:** Matteo Masto, Vincent Favre-Nicolin, Steven Leake, Tobias Schülli, Marie-Ingrid Richard, Ewen Bellec

**Affiliations:** a ESRF, The European Synchrotron, 71 Avenue des Martyrs, Grenoble, France; b Univ. Grenoble Alpes, Grenoble, France; c Univ. Grenoble Alpes, CEA Grenoble, IRIG, MEM, NRS, 17 Avenue des Martyrs, Grenoble, France; DESY, Hamburg, Germany

**Keywords:** Bragg coherent diffraction imaging, image inpainting, deep learning

## Abstract

A deep-learning algorithm is proposed for the inpainting of Bragg coherent diffraction imaging (BCDI) patterns affected by detector gaps. These regions of missing intensity can compromise the accuracy of reconstruction algorithms, inducing artefacts in the final result. It is thus desirable to restore the intensity in these regions in order to ensure more reliable reconstructions. The key aspect of the method lies in the choice of training the neural network with cropped sections of diffraction data and subsequently patching the predictions generated by the model along the gap, thus completing the full diffraction peak. This approach enables access to a greater amount of experimental data for training and offers the ability to average overlapping sections during patching. As a result, it produces robust and dependable predictions for experimental data arrays of any size. It is shown that the method is able to remove gap-induced artefacts on the reconstructed objects for both simulated and experimental data, which becomes essential in the case of high-resolution BCDI experiments.

## Abstract 2

Large pixel detectors exhibit gaps which affect data analysis because of missing data, *e.g.* for coherent diffraction imaging (CDI). A patching-based deep-learning algorithm is proposed, which allows the missing data to be estimated for arbitrary detector dimensions when applied to Bragg CDI, thus reducing the gap-induced artifacts in the reconstructions.

## 1. Introduction

Coherent diffraction imaging (CDI) is a lens-less technique that exploits scattering of a coherent X-ray beam to study nanoparticles with high spatial resolution (Miao *et al.*, 2000 ▸, 2001 ▸; Pfeifer *et al.*, 2006 ▸). The resulting diffraction pattern contains information about the 3D electron-density distribution in the material. However, since the phase information is lost during the measurement, computer algorithms are needed to reconstruct the real-space objects. For the Bragg coherent diffraction imaging (BCDI) technique, iterative algorithms based on the original Gerchberg–Saxton algorithm (Gerchberg, 1972 ▸) are mainly used. This procedure, referred to as phase retrieval (PR), normally entails alternated projections between direct and reciprocal space, and the application of constraints in both spaces such that the algorithm converges towards the solution (Fienup, 1978 ▸; Fienup & Wackerman, 1986 ▸; Favre-Nicolin *et al.*, 2010 ▸; Marchesini, 2007 ▸; Miao *et al.*, 2012 ▸).

In the case of crystalline samples, BCDI allows one to measure the internal strain of particles ranging in size from a few micrometres to 20 nm (Robinson & Harder, 2009 ▸; Richard *et al.*, 2022 ▸; Hofmann *et al.*, 2017 ▸). BCDI enables *in situ* observation of the strain evolution of nanoparticles during temperature variations (Pfeifer *et al.*, 2006 ▸; Harder *et al.*, 2007 ▸; Chatelier, 2024 ▸), during gas reactions (Watari *et al.*, 2011 ▸; Car­nis *et al.*, 2021 ▸; Dupraz *et al.*, 2022 ▸) and in an electrochemical environment (Ulvestad *et al.*, 2014 ▸; Hua *et al.*, 2019 ▸; Atlan *et al.*, 2023 ▸). These reactions often occur at the surface of the nanoparticles; thus a good spatial resolution is required in order to follow their evolution by monitoring the effects at the particles’ surfaces. Since the measured intensity (**I**) corresponds to the squared modulus of the object Fourier transform (FT), the real-space resolution is inversely proportional to the extent of the recorded diffraction pattern. Consequently, there is a requirement for detectors with large sensing areas to achieve high resolution (Bond & Cahn, 1958 ▸).

Standard photon-counting detectors are usually assembled out of pixelated chips separated by insensitive gaps. These gaps consist of a-few-pixel-wide lines, whose size varies according to the detector model. For example, the photon-counting MAXIPIX detector contains a cross-shaped 6 pixel-wide gap (Ponchut *et al.*, 2011 ▸) while the Eiger2M detector (Johnson *et al.*, 2014 ▸), having a larger sensing area, has both 12 and 38 pixel-wide gaps. Technological solutions are on the horizon, *e.g.* the PIMEGA or through-silicon via technology (Campanelli *et al.*, 2023 ▸), but the majority of pixel detectors available at the time of writing have gaps between active areas.

A 3D BCDI pattern is obtained by rotating the sample and by stacking each 2D detected image for each rotation angle. This implies that the gap lines in each 2D image turn into gap planes of empty pixels in the full 3D pattern.

The effect of these regions of missing intensity on the recorded diffraction is the corruption of the PR algorithms, which eventually leads to the presence of artefacts in the reconstructed real-space object (Carnis *et al.*, 2019 ▸) (see Fig. S1 in the supporting information). These artefacts become more significant when the BCDI 3D array is large, thus severely limiting the reconstructed object resolution. This phenomenon is even more problematic in CDI where the near-forward information is lost due to the presence of the beamstop. Some solutions have been proposed, such as normalization of the reconstructed direct beam intensity using the measured part of the diffraction array (Nishino *et al.*, 2003 ▸), using multiple measurements (Steinbrener *et al.*, 2010 ▸) or via a maximum-likelihood estimation (Barmherzig & Sun, 2022 ▸). In BCDI, the usual method is to not apply the reciprocal-space modulus constraint on the gapped areas (Favre-Nicolin *et al.*, 2020 ▸).

Here, we propose to preprocess the 3D experimental BCDI data affected by these gaps using a deep-learning (DL) inpainting method. Our model is able to make consistent predictions of the in-gap intensity on experimental BCDI data, thus reducing artefacts in the reconstructed object.

Image inpainting has been widely explored in the field of photography and image processing for the restoration of damaged pictures (Bertalmio *et al.*, 2000 ▸; Elharrouss *et al.*, 2019 ▸). Many techniques have been developed, from classical polynomial interpolations to more advanced techniques such as diffusion-based methods or sparse representation methods (Jam *et al.*, 2021 ▸). More recently, image inpainting has been addressed with the use of deep convolutional neural networks which have shown promising and accurate results in different fields (Xiang *et al.*, 2023 ▸).

In the field of CDI, and more specifically BCDI, DL methods have been exploited for defect identification, classification (Lim *et al.*, 2021 ▸; Judge *et al.*, 2022 ▸) and PR (Cherukara *et al.*, 2018 ▸; Chan *et al.*, 2021 ▸; Yao *et al.*, 2022 ▸; Wu *et al.*, 2021*a* ▸,*b* ▸). Image inpainting for X-ray diffraction has already been studied both with classical methods (Liu *et al.*, 2017 ▸) and more recently with DL algorithms by Bellisario *et al.* (2022 ▸) on 2D simulated data and by Chavez *et al.* (2022 ▸) on 2D X-ray scattering images. In the case of DL approaches for PR, an unsupervised fine-tuning procedure can be used to refine the reconstructed object (Yao *et al.*, 2022 ▸) or even a fully unsupervised method, leading to a reconstruction without the need for a large training data set (Wu *et al.*, 2021*b* ▸). However, in our case of DL for gap inpainting, we cannot train our model in an unsupervised fashion; hence we choose instead a supervised approach. Nevertheless, a recurrent problem in supervised DL for BCDI is the lack of a large experimental data set, thus the need to train the model using mostly simulated diffraction data. This limitation often biases the DL models, which eventually yield poor results on experimental data. Moreover, these DL models use a fixed input–output size. This is inconvenient for practical use since typical experimental BCDI data are cropped and centred during preprocessing, leading to a possible different array size each time.

Here, we propose a solution that solves both the limited experimental data set and the size constraint issues for the case of detector gap inpainting through the implementation of a ‘patching model’ trained on small 32 × 32 × 32 pixel-size cropped portions (**P**) of the diffraction patterns. This patching technique allows for the use of a large number of small portions from experimental BCDI data along with simulated ones. Henceforward, we can use a much lighter and rapidly trainable DL network. Our model can then be applied on a large BCDI 3D array, regardless of the data size. The size of **P** was chosen such that it was larger than the usual gap size and the finite size oscillations of the intensity pattern.

## 2. Results

### 2.1. Data set preparation

The data set used to train the model contains a mix of simulated and experimental Bragg coherent diffraction patterns. Experimental data (ED) were taken from measurements performed at the ID01 beamline of the European Synchrotron (ESRF, in Grenoble, France) (Leake *et al.*, 2019 ▸). These measurements were performed during different beamtimes and correspond to (i) Pt particles dewetted on sapphire and YSZ (yttria–zirconia), having a Winterbottom shape measured at several temperatures and gas conditions, (ii) Pd and PdCe on glassy carbon, with a Wulff shape in an electro­chemical environment, following hydrogen loading, (iii) Ni particles on sapphire during CO_2_ adsorption, and (iv) cubic CaCO_3_ particles on glassy carbon. Simulated data (SD) have been constructed following the procedure described by Lim *et al.* (2021 ▸), *i.e.* creating 3D face-centred cubic crystals from random atomic elements, crystal shapes and sizes via *MERLIN* (Rodney, 2010 ▸) and simulating the energy relaxation using *LAMMPS* (Plimpton, 1995 ▸). The 128 × 128 × 128 pixel-size BCDI diffraction pattern of the 200 Bragg peak was then calculated using the *PyNX* package (Favre-Nicolin *et al.*, 2020 ▸) with random particle orientation and reciprocal-space step size, *i.e.* a different oversampling ratio. However, the resulting SD are still very different from what was measured experimentally and could bias our DL model, diminishing its applicability to experimental data. To prevent this, we modified the SD by introducing noise in both reciprocal and real space as detailed in Section S2 in the supporting information.

**Figure 1:**
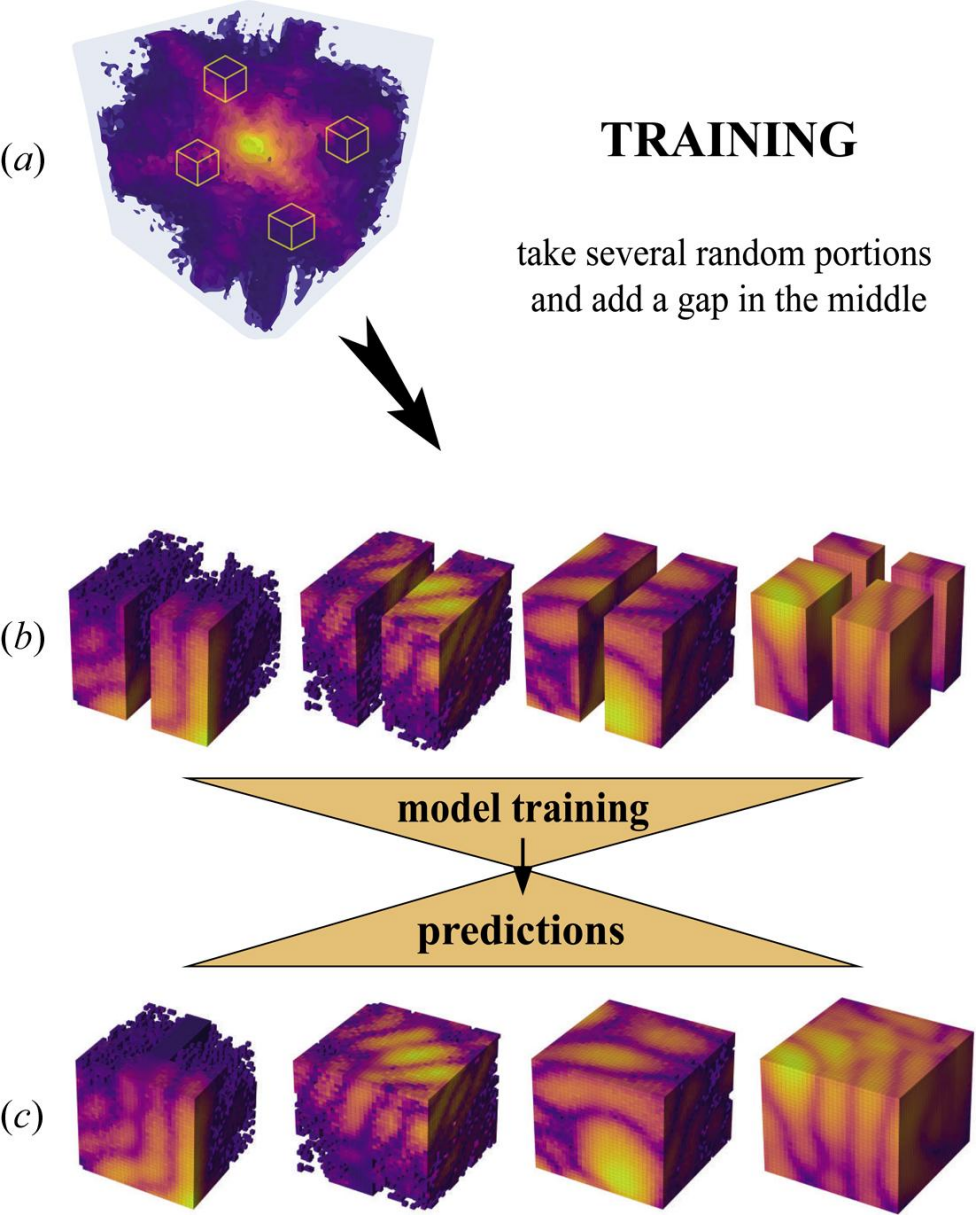
Sketch of the data processing and DL model training. (*a*) Large Bragg coherent diffraction pattern where small 32 × 32 × 32 pixel-size portions are randomly selected. (*b*) Small portions in renormalized logarithmic scale, artificially masked with zeros to simulate detector gaps and used as input to the DL model. (*c*) DL model predictions for the corresponding masked inputs with the inpainted gap.

From each diffraction pattern, 10 portions **P** of 32 × 32 × 32 pixel size have been cropped out pseudo-randomly (see Fig. 1 ▸). Having noticed poorer accuracy for the prediction around low-intensity regions, we preferentially selected portions from peripheral areas over the centre of the peak. Thus the final data set, composed of 50% ED and 50% SD, contains 30 000 of these small portions.

### 2.2. Data preprocessing

During the DL model training, an artificial vertical mask of zero-intensity pixels, and of fixed size, was added in the middle of each single portion **P**, as defined above, in order to simulate the presence of the detector gap [Fig. 1 ▸(*b*)]. To include the case of cross-shaped gaps, an additional mask, of equivalent size, was applied horizontally at a random position to a certain subset of the training data [see the last example in Fig. 1 ▸(*b*)].

The last preprocessing step transforms the data to a logarithmic scale and normalizes each image between 0 and 1 in order to avoid overfitting high-intensity regions over the low-intensity ones. The masked images were then used as model input while the ground-truth unmasked images were used in the calculation of the loss function, as a comparison with the DL predictions.

### 2.3. Network architecture and training

**Figure 2:**
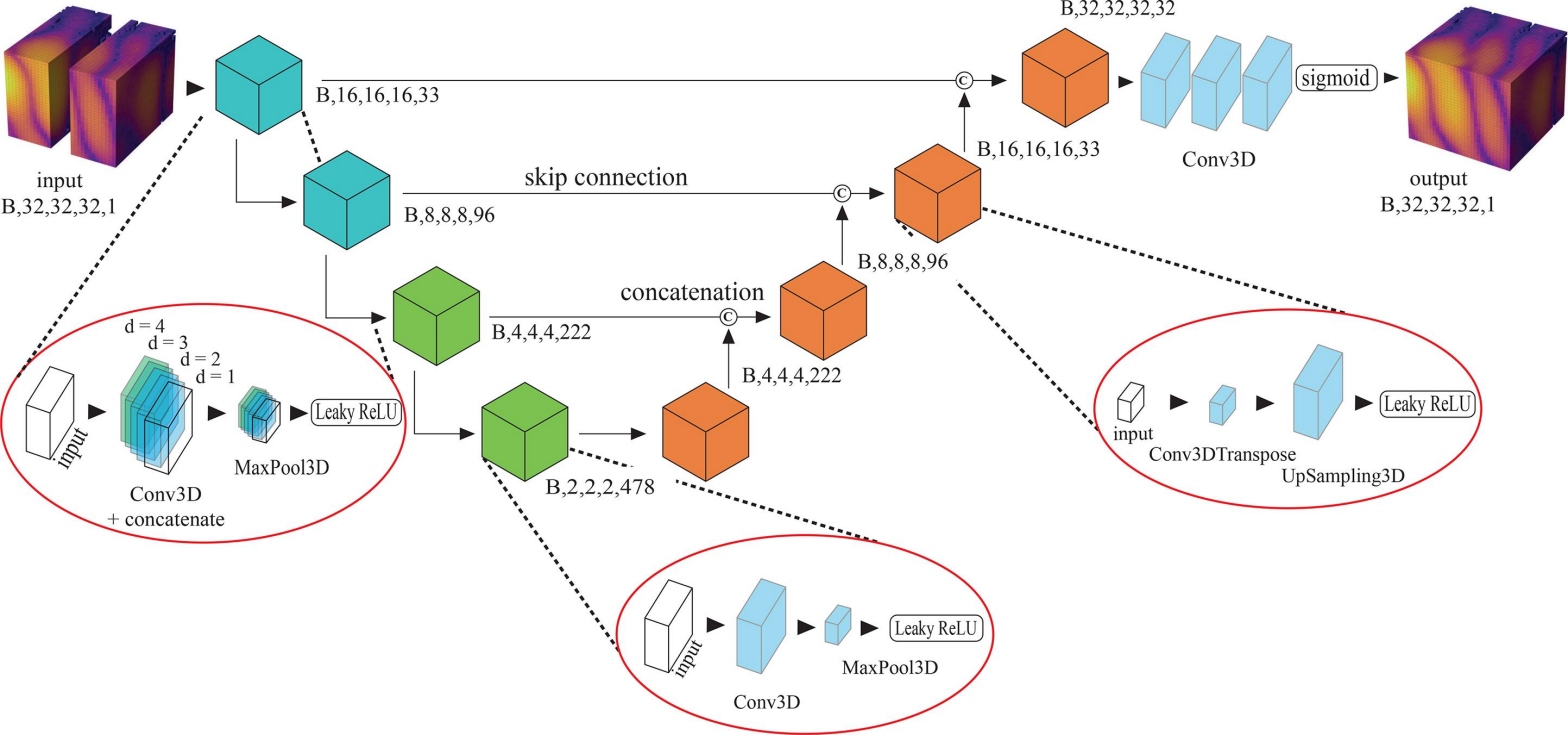
Architecture of the DL model. It is based on a modified U-Net architecture with the use of dilated convolutions in the first two encoder blocks (left red circle) where the input was concatenated to its convolutions with different dilation rates (*d* parameter) before the MaxPooling layer. The gap-affected small portions **P**, in batches (B) of 32, were used as input (top left) and progressively sent through the encoder section down to a small volume of 2 × 2 × 2 pixels. Each building block of the decoder section (orange cubes) takes as input the concatenation of its previous block’s output with the corresponding output of the encoder block of the the same size. The final output (top right) is a batch of inpainted **P**.

The adopted DL model was based on the U-Net architecture (Ronneberger *et al.*, 2015 ▸) (see Fig. 2 ▸). The choice of U-Net was corroborated by many successful studies that have used it for image-to-image processing and inpainting specifically (Siddique *et al.*, 2021 ▸; Ozturk, 2020 ▸; Yan *et al.*, 2018 ▸; Chavez *et al.*, 2022 ▸; Bellisario *et al.*, 2022 ▸). It consists of two main blocks, namely the encoder and the decoder. The encoder is composed of four convolutional layers followed by MaxPooling and leads to a reduction of the data array size from 32 × 32 × 32 to 2 × 2 × 2. The decoder section uses other convolutional and UpSampling layers to enlarge the array back to its original size. Information is transferred between each encoder and decoder layer through skip connections, ensuring an easier search for the loss function’s absolute minimum (Li *et al.*, 2017 ▸). Skip connections were found to be particularly effective because of the strong similarity between input and output images. All encoder and decoder layers use the Leaky ReLU activation function with a slope of 0.2 for negative inputs. Finally, three additional convolutional layers were added after the decoder as a way to avoid image smoothing by the array expansion in the decoder. The sigmoid activation function is used as the last layer in order to bound the output values between 0 and 1, for the model outputs to have the same intensity range as the inputs. To improve the receptive field of the first convolutional layers and thus provide higher long-range correlation understanding in the feature extraction, dilated convolutions have been employed with variable dilation rate (Chen *et al.*, 2017 ▸). More precisely, as depicted in Fig. 2 ▸, in the first two encoder blocks the input was concatenated with four different convolutions of itself, each one with a different dilation rate (the *d* parameter in Fig. 2 ▸). Standard convolutional layers were used in the last two encoder blocks as the size of the inputs was already small enough to be treated with normal convolutional layers and a 3 × 3 × 3 pixel-size kernel.

The network was built using the *Tensorflow* Python library (Abadi, 2016 ▸) and was trained for 100 epochs, with the ADAM optimizer (Kingma & Ba, 2017 ▸) starting with a learning rate of 10^−3^ and decreasing progressively using the ReduceLROnPlateau callback available in *Tensorflow*. The shuffled data set of 30 000 small portions was split into training (93.5%), validation (4%) and test (2.5%) sets. Batches of 32 images were used, and training and validation losses were monitored at each epoch in order to avoid overfitting to the training data set. Inpainted output and ground-truth regions were compared using a custom loss function consisting of the sum of three main terms, namely a mean absolute error (MAE), a structural similarity index perceptual loss (Wang *et al.*, 2004 ▸) and an MAE on the image gradients.

To assess the DL model performance we used the Pearson correlation coefficient (PCC). This coefficient measures the linear correlation between two images and in our specific case yields an estimation of the similarity between the DL prediction and the corresponding ground truth; it is thus an indication of the predictive accuracy of the model. It is defined by
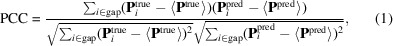
where **P**^true^ is the 32 × 32 × 32 pixel-size ground-truth portion in logarithmic scale without a gap and **P**^pred^ is the same portion where the gap region was inpainted using our DL model. The 〈〉 symbol corresponds to the average over the gap. We note that the PCC for identical images was equal to 1.

Table 1 ▸ shows the average PCC values over a batch of 1000 samples of small portions from experimental BCDI data (where gaps have been artificially added). Vertical gaps of different sizes are considered. As expected, the accuracy decreases when the gap size increases since the prediction of the in-gap fringes becomes more and more difficult. Examples of DL predictions on small BCDI regions with a 6 pixel gap are shown in Figs. S7 and S8 for, respectively, SD and ED, demonstrating accurate in-gap intensity prediction.

### 2.4. Results in reciprocal space

In order to make a prediction of the in-gap intensity of a large 3D BCDI array of arbitrary size, we use a ‘patching’ method. A 32 × 32 × 32 pixel-size portion **P** centred around the gap of the large image was used as the DL model input and the in-gap intensity was predicted in this region. **P** was then repeatedly shifted by 1 pixel at a time along the gap and the prediction was calculated again, until the whole gap intensity was reconstructed. The final step involves averaging the overlapping predicted pixels, which contributes to robust DL predictions even when applied to ED. This averaging process helps mitigate potential prediction errors by smoothing them out.

However, this method can be time consuming as a prediction for a cross-shaped gap on 128 × 128 × 128 pixel-size BCDI data can take up to 12 min. To speed up this process, one can shift **P** by more than 1 pixel at a time, drastically decreasing the prediction time to 1 m 30 s for 4 skipped pixels, without significantly worsening the accuracy. More details on this skip method are available in Section S5 of the supporting information.

**Figure 3:**
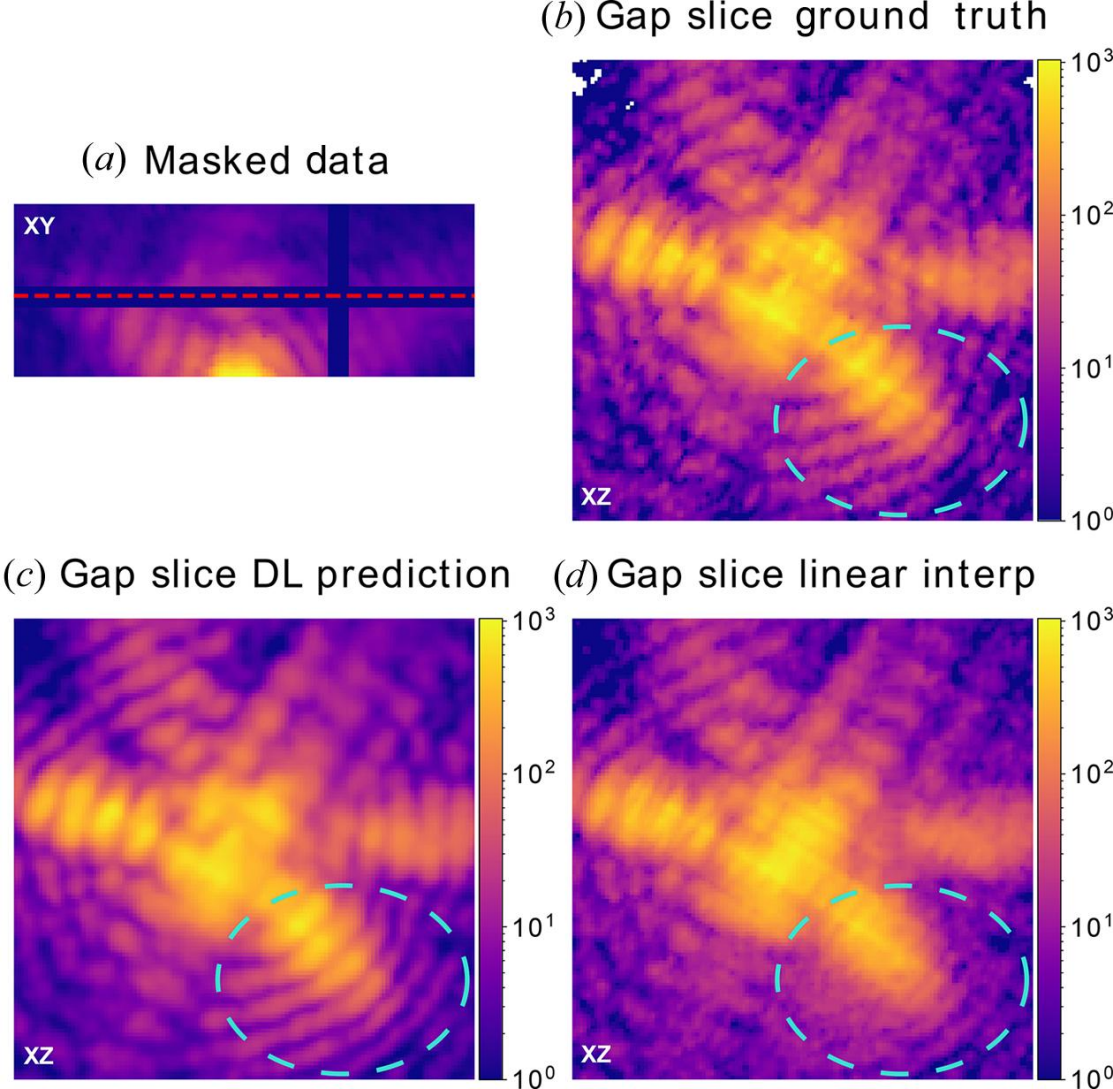
In-gap slice comparison between DL prediction and standard interpolation on experimental BCDI data. (*a*) 3D experimental BCDI data masked with a cross-like 6 pixel-wide gap – the red dashed line indicates the location of the perpendicular ‘in-gap slices’ shown in (*b*), (*c*) and (*d*). In-gap slice (*b*) ground truth, (*c*) DL model prediction and (*d*) standard linear interpolation using pixels immediately around the gap. Only our DL model was able to restore the correct fringe pattern as highlighted by the turquoise dashed circles.

In order to test the DL model accuracy on large BCDI data arrays, we use the patching method on a large experimental BCDI array where a 6 pixel-wide cross-shaped gap was added. Fig. 3 ▸(*a*) displays the position of the gap in the *XY* plane while Fig. 3 ▸(*b*) shows the ground-truth in-gap intensity in the *XZ* plane. Our DL model prediction is shown in Fig. 3 ▸(*c*), where the fringe pattern was accurately reproduced. The ‘grainy’ features of the ground truth are not reproduced due to the intrinsic denoising effect induced by the model training process (Krull *et al.*, 2019 ▸).

As a comparison, a standard linear interpolation (LI) is shown in Fig. 3 ▸(*d*). The cubic and nearest-neighbour interpolations are illustrated in Fig. S9.

An improvement of the result from the DL model with respect to standard interpolation algorithms was observed in all the cases, in particular when comparing the fringe patterns in the bottom left of Figs. 3 ▸(*c*) and 3 ▸(*d*). Where LI fails to reproduce the oscillations, DL succeeds. This was expected, since standard interpolation algorithms have no *a priori* knowledge of the oscillatory nature of diffraction fringes.

**Figure 4:**
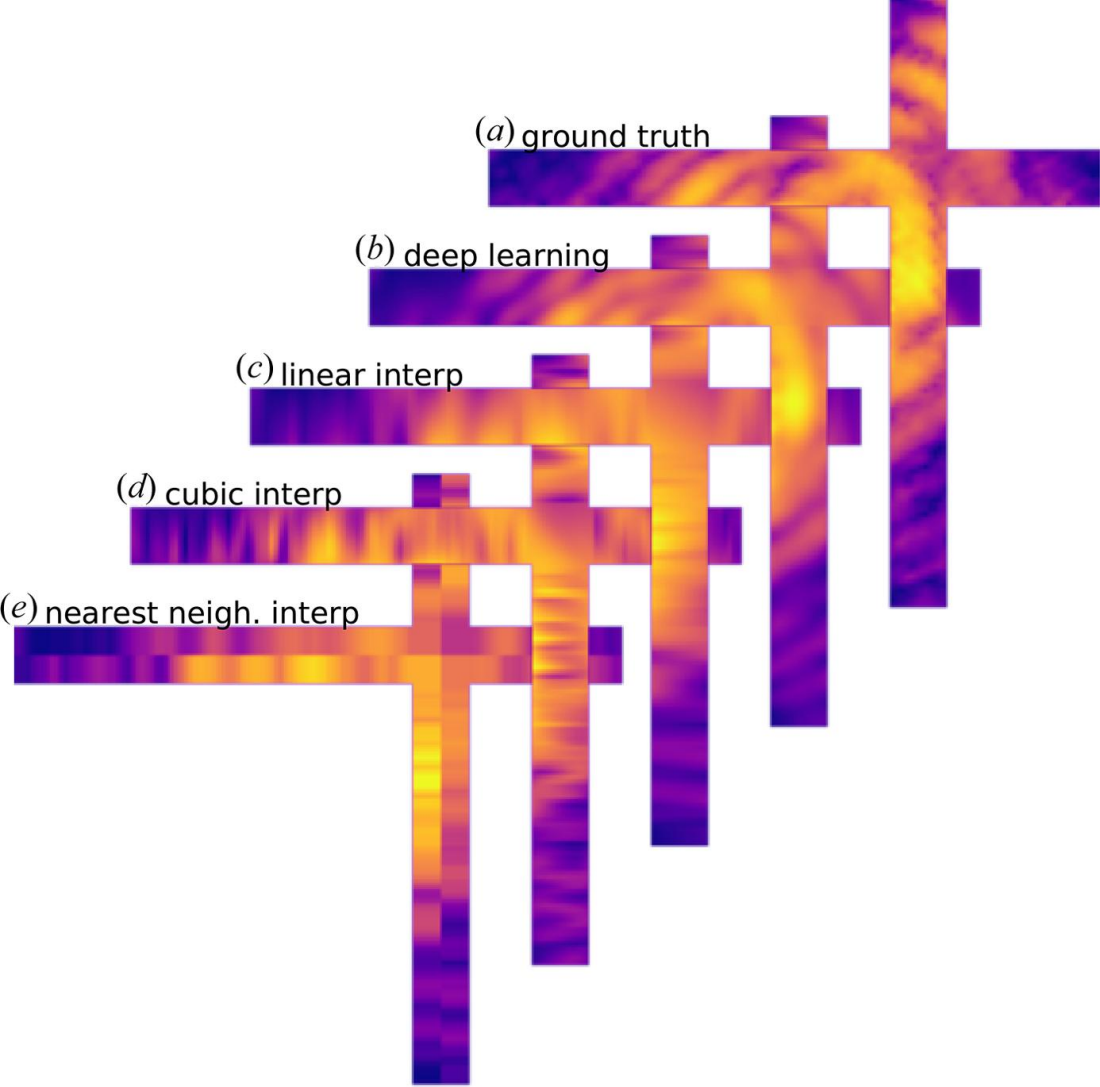
Comparison between DL prediction and several standard interpolations across the gap. (*a*) Ground-truth intensity in the 12 pixel-wide cross-shaped gap (only the gap area is shown). (*b*) DL prediction. (*c*), (*d*), (*e*), respectively, linear, cubic and nearest-neighbour interpolation. Only the DL model was able to recover the accurate fringe curvature across the gap.

This is further demonstrated in Fig. 4 ▸ where the in-gap intensity is shown in the *XY* plane for a gap size of 12 pixels. The DL algorithm [Fig. 4 ▸(*b*)] was able to predict the correct fringe curvature across the gap. On the other hand, the standard interpolations [see Figs. 4 ▸(*c*)–4 ▸(*e*)] neglect this curvature and reproduce straight oscillations perpendicular to the edges of the gap region.

### 2.5. Performance assessment

**Figure 5:**
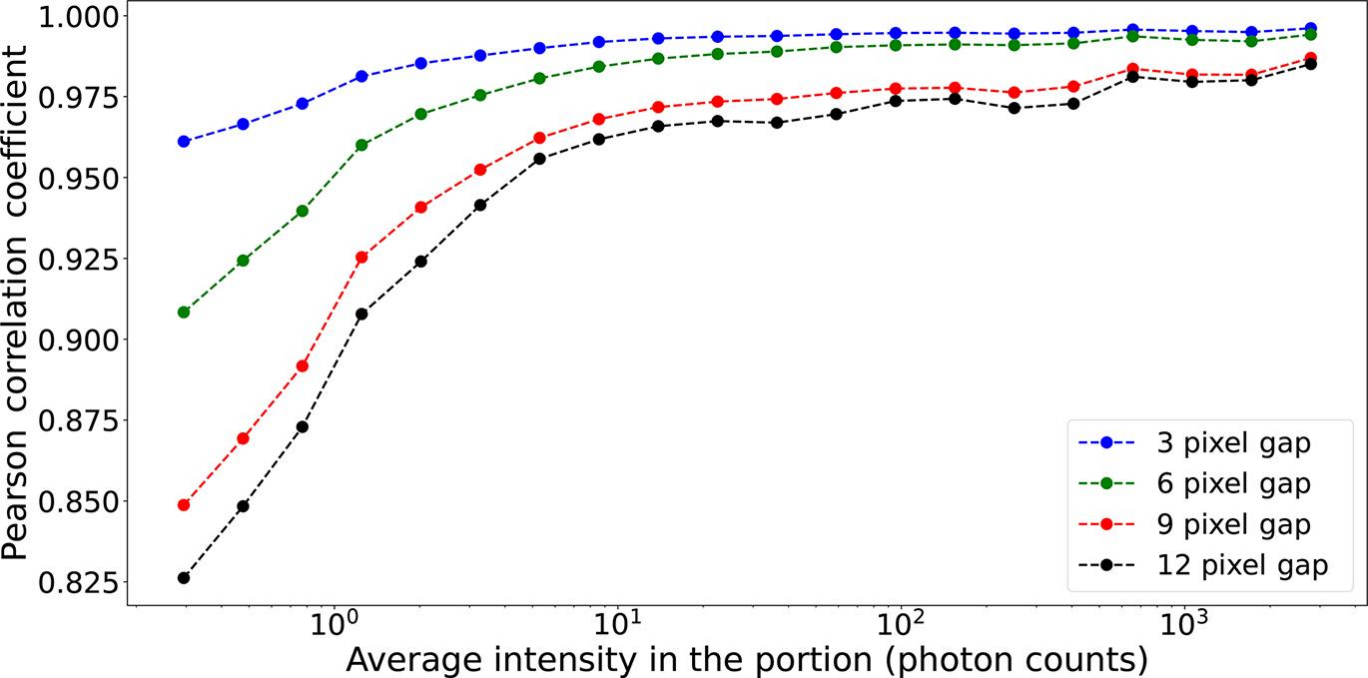
Prediction accuracy versus average intensity in a cropped portion. The model prediction becomes more accurate as the overall intensity inside the considered portion increases. Conversely, in cases of low photon counts – indicating a prevalence of noise within the portion – the predictions were more prone to inaccuracies.

In this section we discuss the accuracy of our model with respect to (i) the amount of intensity inside the cropped portion **P** and (ii) the oversampling ratio. In order to assess the model accuracy for the first case, we used a 128 × 128 × 128 pixel-size experimental diffraction pattern and we randomly cropped out 1000 portions **P** of 32 × 32 × 32 pixels. A vertical gap was placed in the middle of each **P** and the DL model was used to predict the in-gap intensity. The PCC accuracy as given in Table 1 ▸ was then calculated for each **P** individually and its average is shown as a function of the average photon count in **P** (see Fig. 5 ▸). Lower PCC scores are obtained when the average intensity in the region is smaller, which is expected as the absence of significant features that are lost in the Poisson noise prevents accurate DL prediction. Moreover, as expected, from Fig. 5 ▸ it emerges that the smaller the gap size, the better the accuracy of the prediction.

**Figure 6:**
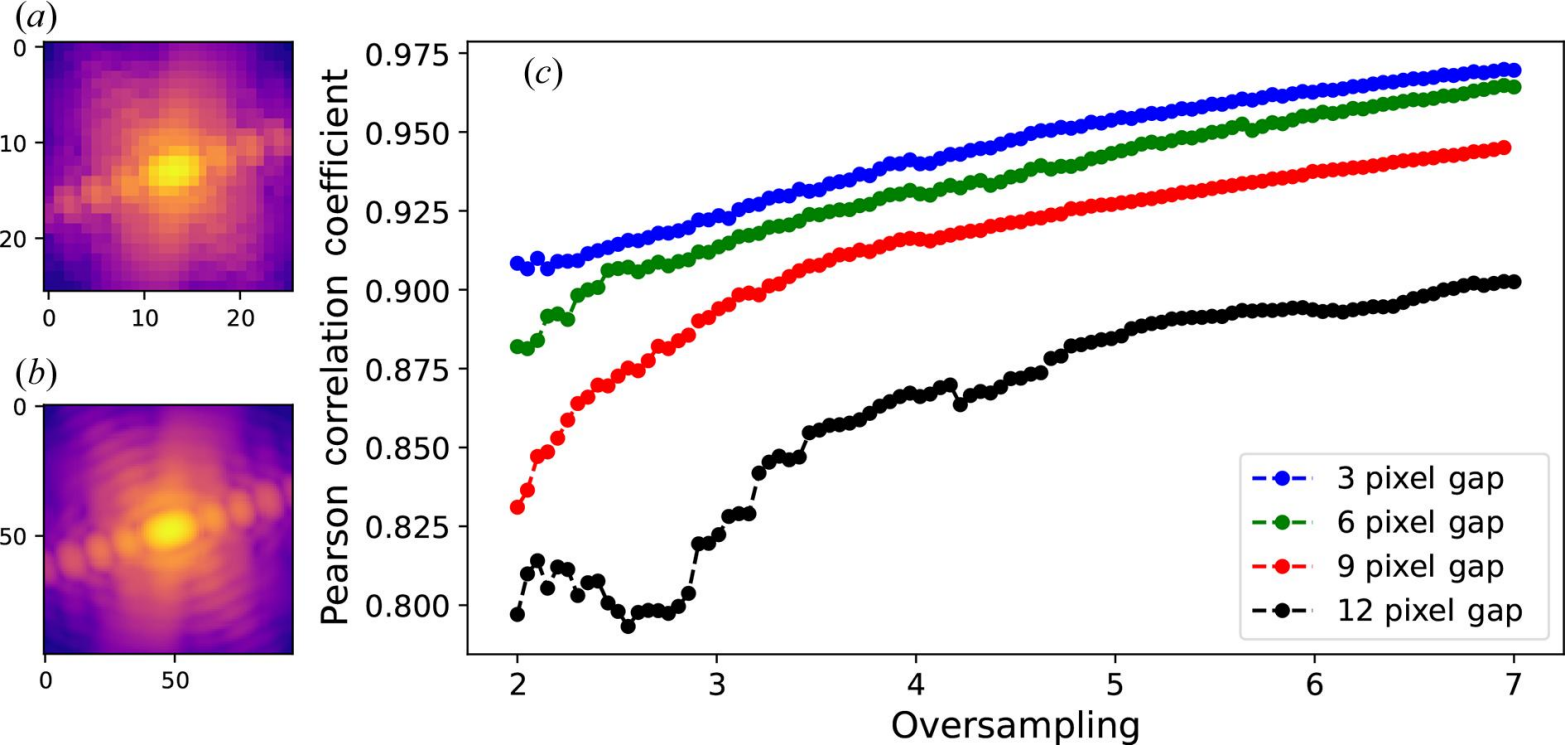
Prediction accuracy versus oversampling. (*a*) Low and (*b*) high oversampling simulated BCDI array of the same region. (*c*) Prediction accuracy as a function of BCDI oversampling ratio for different gap sizes.

In order to compute the model accuracy as a function of the oversampling ratio, we simulate BCDI arrays of the same region for different oversampling ratios (ORs), as shown in Figs. 6 ▸(*a*)–6 ▸(*b*). Since a different OR implies a different array size, comparing the model accuracy is not straightforward. To do so, we make the prediction of the full image using the method illustrated in Fig. S13. For each OR, a vertical gap mask was applied to the whole BCDI array and the DL prediction was calculated. The gap was then shifted and this procedure was repeated until the whole BCDI array was predicted using our model, thus leading to a full BCDI predicted image. The PCC shown in Fig. 6 ▸(*c*) was then calculated using the whole BCDI array for different ORs and model gap sizes. As expected, the predictions are more accurate for large ORs and small gap sizes (*i.e.*, large oscillation periods relative to the gap width). Some prediction examples are given in Fig. S14.

### 2.6. Reconstructions in real space

#### 2.6.1. Simulated real-space result

**Figure 7:**
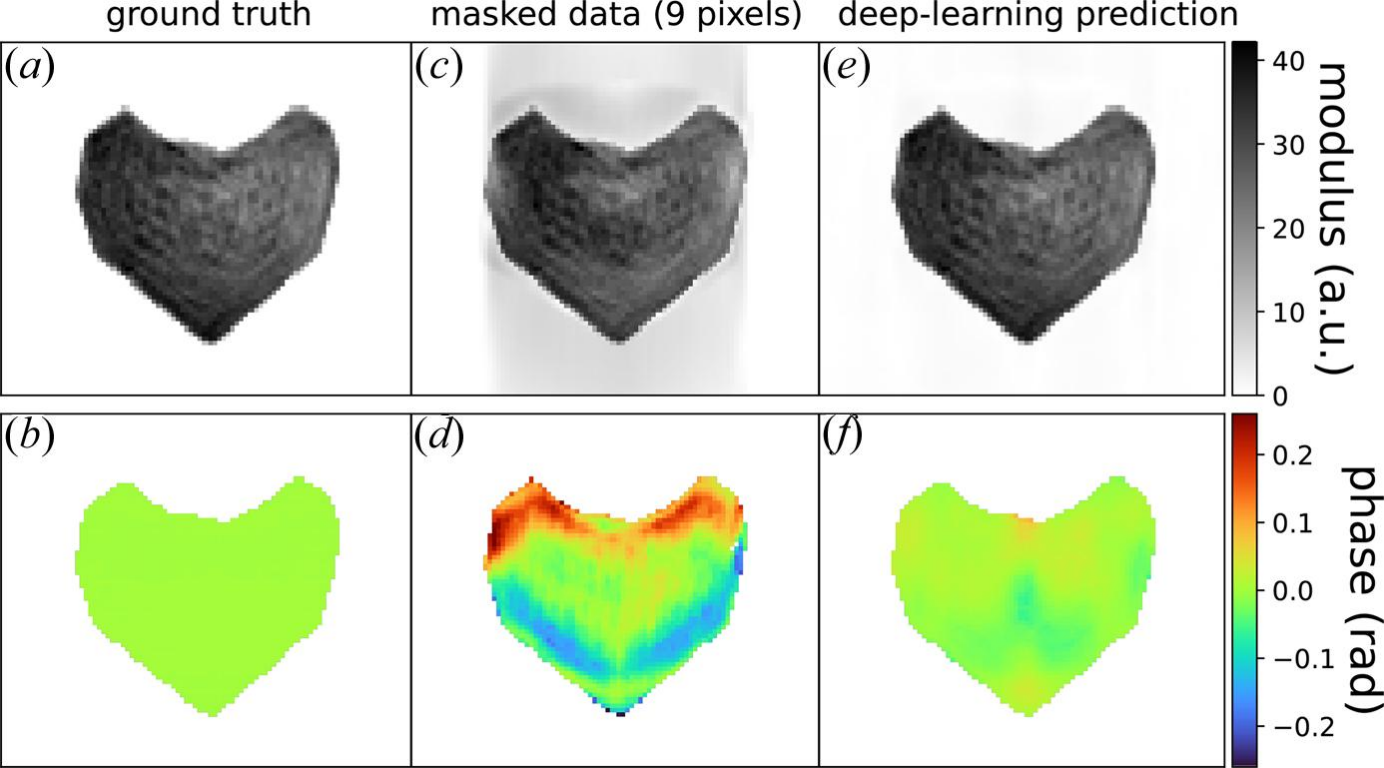
DL inpainting result for real-space object reconstruction. (*a*), (*b*) Central slice of the ground-truth object modulus and phase obtained from a simulated diffraction pattern with no gap. (*c*), (*d*) Reconstruction with a 9 pixel-wide cross-like gap-affected diffraction pattern. (*e*), (*f*) Reconstruction after DL gap inpainting, drastically reducing the artefacts induced by the gap. The corresponding diffraction patterns are available in Fig. S15. Note that in this example the phase of the ground-truth object has been artificially set to zero (in contrast to Fig. 8 ▸) for an easier comparison.

Since the final goal of the BCDI technique, before physical analysis, is the reconstruction of the real-space complex object, we assess here the reconstructed object quality before and after DL gap inpainting. A simulated BCDI array was used, starting from a reconstruction by Carnis *et al.* (2019 ▸). After the reconstruction from the experimental diffraction pattern, the real-space phase of the particle was artificially set to zero [see Figs. 7 ▸(*a*)–7 ▸(*b*)], making the evaluation of the gap effect easier. From this reference ‘ground-truth’ object **O**, the simulated diffracted amplitude corresponds to **A** = FT[**O**], where FT is the Fourier transform.

A 9 pixel-wide cross-shaped gap mask was then applied to **A** [see Fig. S19(*b*)] and the corresponding real-space object was calculated from the inverse FT [Figs. 7 ▸(*c*)–7 ▸(*d*)]. The presence of the gap in the diffraction pattern induces artefacts in real space manifesting as non-zero modulus values outside the support region along the directions perpendicular to the gap planes [Fig. 7 ▸(*c*)]. Most importantly, the gap induces variations in the object phase, and thus the reconstructed displacement field and strain (Godard, 2021 ▸), especially near the sample surfaces [Fig. 7 ▸(*d*)]. Here, a phase variation of ±0.2 radians is observed in Fig. 7 ▸(*d*), resulting in an error of ±7 pm in the lattice displacement field for the 111 Pt reflection. These artefacts are particularly problematic in the cases of (electro-)catalytic experiments (Atlan *et al.*, 2023 ▸) or *in situ* gas experiments (Ulvestad *et al.*, 2016 ▸; Kim *et al.*, 2018 ▸; Abuin *et al.*, 2019 ▸; Kawaguchi *et al.*, 2019 ▸; Dupraz *et al.*, 2022 ▸), where the chemical reactions occur at the nanoparticle’s surfaces and can be studied by following the strain evolution in these regions. The presence of a large gap, or a gap close to the centre of the Bragg peak, could lead to a physical misinterpretation from a poorly reconstructed phase.

Afterwards, our DL model was used to predict the in-gap masked intensity [see Fig. S15(*c*)], and the corresponding object was computed via the inverse FT using the ground-truth reciprocal-space phase [Figs. 7 ▸(*e*)–(*f*)]. The artefacts on the reconstructed modulus disappear almost entirely. Furthermore, the reconstructed phase standard deviation is five times lower than that calculated for the case with a gap and does not present any large variations close to the surfaces, showing that our DL method is suitable for the object reconstruction.

Referring to the work of Carnis *et al.* (2019 ▸), we evaluate the root-mean-squared error (RMSE) values of the strain for the particle in Fig. S18 for different gap sizes. The wider the gap, the larger the variation from the mean, and hence the less precise the obtained strain distribution. However, it is clearly visible from the same figure that the restoration of the diffraction intensity using our DL method significantly reduces the error on the strain calculation. Also note that the mean value of the strain obtained from masked diffraction patterns differs from the expected zero, as depicted in Fig. S17.

#### 2.6.2. Experimental real-space result

In order to obtain a nanoparticle reconstruction with high spatial resolution, one generally has to measure a relatively large BCDI array. With typical photon-counting detectors, this leads to a region with a large gap, as shown in Fig. S1(*a*). One common solution is to run the PR algorithms leaving the in-gap pixels free.

**Figure 8:**
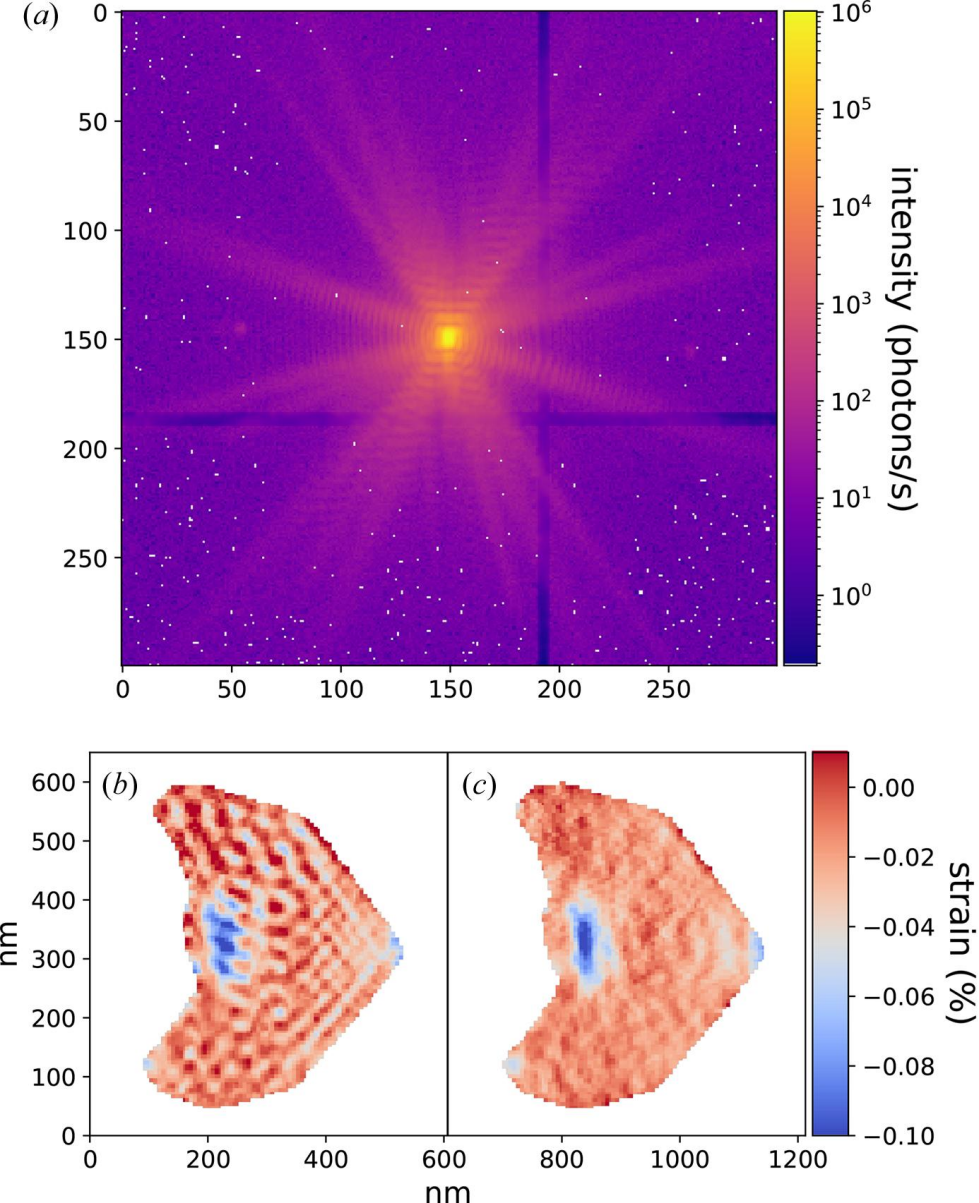
DL inpainting result for high-resolution ED containing a real gap. (*a*) DL inpainted high-resolution BCDI experimental array containing a real gap. (*b*) Object strain reconstruction leaving in-gap pixels free during PR. (*c*) Reconstructed strain using DL inpainting. The strong oscillation artefacts visible in (*b*) are removed by the DL inpainting.

However, with this approach PR algorithms often overestimate the intensity distribution inside the gap, leading to strong oscillation artefacts in the phase and strain of the reconstructed object, as shown in Fig. 8 ▸(*b*).

On the other hand, by inpainting the gap with our DL model before the PR [Fig. 8 ▸(*a*)], the resulting strain map in Fig. 8 ▸(*c*) does not show any of these artefacts, indicating that the in-gap prediction is accurate. Furthermore, we tested other methods for comparison, namely leaving pixels free only at the streak position and setting the in-gap intensity to 0. The results are depicted in Figs. S19–S21 and show that DL inpainting is the best way to obtain a reliable high-resolution reconstruction from BCDI data with gaps. A second high-resolution example is shown in Fig. S22.

### 2.7. Fine-tuning

**Figure 9:**
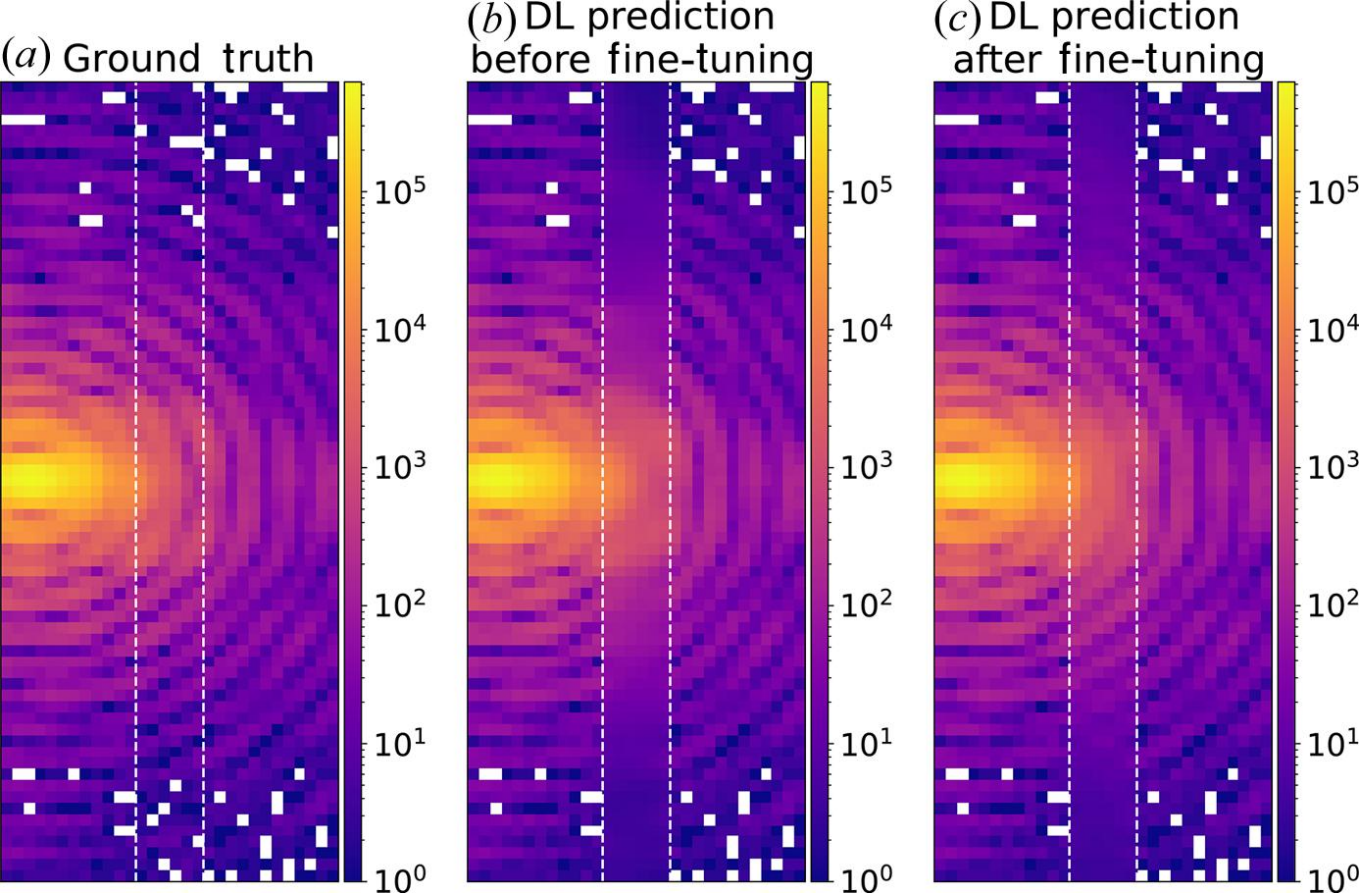
Fine-tuning. (*a*) The central slice of an experimental diffraction pattern in logarithmic scale. An artificial 6 pixel-wide vertical gap was added in the region between the two white dashed lines. (*b*) The corresponding slice after DL inpainting where the fringe pattern is not correctly retrieved. (*c*) The same slice of the inpainted image after 2 epochs of fine-tuning of the DL model. The fringe pattern was more reliably recovered.

There may be cases for which the DL model does not yield satisfactory predictions inside the gap, such as when the target image is too different from the training data set, as shown in Fig. 9 ▸(*b*). To overcome these situations, it is possible to fine-tune the model using a specific data set obtained only from the target image. Our approach involves a secondary short training phase for the model, conducted on a limited data set (6400 portions) derived from a random sub-sampling of the same 3D diffraction pattern affected by gaps that we aim to restore. This training exclusively uses portions of the detector that remain unaffected by gaps. The second training occurs typically within 2 to 5 epochs and usually takes up to 1 or 2 min. By performing this fine-tuning, the model is biased on purpose to operate with specific features of the image of interest (oversampling, particle shape, detector *etc.*), thus improving the performance of the real gap prediction [see Fig. 9 ▸(*c*)]. We emphasize that this fine-tuning procedure is only advised when the prediction obtained with the pre-trained model is relatively poor.

## 3. Conclusion

In the present work, a DL-based approach for inpainting 3D BCDI arrays affected by a detector gap is proposed. The key point of our method is the use of a ‘patching’ technique where only small portions of the BCDI arrays are used during training. This technique offers several benefits: (i) it effectively removes the constraint on the size of the BCDI array, meaning that there is no need to train different models for different array sizes; (ii) given the small volume of the portions, the training of the model is faster; and most importantly (iii) cropping small portions of a large data array leads to a drastic increase in the amount of experimental data available during the training, removing possible biases that often occur using only a simulated data set. Our model achieves high accuracy on experimental BCDI data and was able to remove possible reconstruction artefacts on the real-space object, especially in the case of high-resolution BCDI data.

This DL ‘patching’ approach could be applied to other imaging techniques that are associated with missing pixel problems and a lack of large experimental training data sets, such as CDI, ptychography or any other techniques with image spatial correlations.

## 4. Code availability

The codes for this study are available and accessible via the link https://github.com/matteomasto/Patching_DL.

## Supplementary Material

Supporting information including supplementary figures. DOI: 10.1107/S1600576724004163/yr5131sup1.pdf

## Figures and Tables

**Table 1:** Average DL model accuracy on 32 × 32 × 32 pixel-size small BCDI portions over a batch of 1000 samples The accuracy decreases as the gap size increases.

Gap size (pixels)	3	6	9	12
Pearson correlation coefficient	0.989	0.977	0.955	0.946
