# Supplementary material for: Patching-based deep-learning model for the inpainting of Bragg coherent diffraction patterns affected by detector gaps
Source: J Appl Crystallogr. 2024 Jun 18;57(Pt 4):966–74. doi: 10.1107/S1600576724004163 (PMC11299604; doi:10.1107/S1600576724004163)
Supplement: Supplementary file 1 [file j-57-00966-sup1.pdf]

JOURNAL OF APPLIED CRYSTALLOGRAPHY

Volume 57 (2024)

Supporting information for article:

Patching-based deep-learning model for the inpainting of Bragg coherent diffraction patterns affected by detector gaps

Matteo Masto, Vincent Favre-Nicolin, Steven Leake, Tobias Schülli, Marie-Ingrid Richard and Ewen Bellec

S1. GAP PROBLEM IN BCDI

Supplementary Fig. 1**a** shows a typical example of Bragg Coherent Diffraction Imaging (BCDI) data having a detector gap issue. Figures **b-c** show the reconstructed object modulus and strain respectively. Despite the gap representing only 4% of the BCDI array and being relatively far from the peak centre, visible oscillation artefacts are observed on the reconstructed object. This gap problem prevents reliable high-resolution BCDI reconstructions.

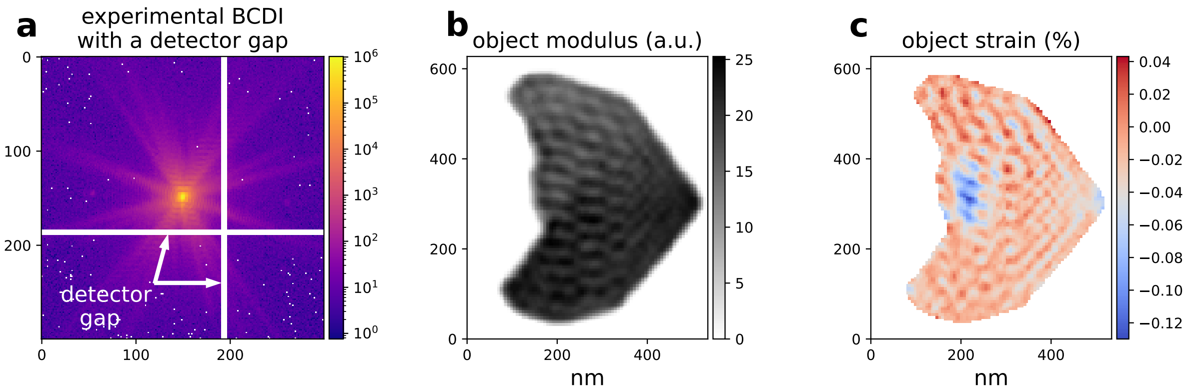

Fig. 1: Gap problem in BCDI. a BCDI array with gapped region. **b** Reconstructed object modulus and **c** strain. Oscillations artefacts induced by the gap presence are visible.

S2. SIMULATION OF REALISTIC BCDI DATA

S2.1 Differences between simulation and experimental BCDI data

Simulated BCDI dataset were created with the same method as in *Lim et al*[1]. First, perfect crystal atomic positions are created using MERLIN[2], having different particles shapes (Wulff, Winterbottom, octahedron, cubic) with random set of parameters including particle size, shape, surface ratios and atomic elements. These particles are then relaxed using LAMMPS[3] to obtain a realistic strain distribution. The corresponding reciprocal space complex amplitude array $\mathbf{A}$ is then calculated with the kinematic sum using PyNX (see Ref.[4]). Different oversampling ratios[5] have been considered. The corresponding BCDI array used to train our model is given by $\mathbf{I} = |\mathbf{A}|^2$.

However, the resulting intensities $\mathbf{I}$ are still very different from real experimental data as shown in Supplementary Fig. 2 where both the projection (**a**) and central slice (**c**) are compared with the ones of a real experimental BCDI array having a similar oversampling ratio (see Supplementary

Fig. 2**b** and **d**). First, the experimental data looks more "roundish" than simulated data (compare **a** and **b**). This is due to the fact that our simulated particles have very well-defined facets with sharp corners, which is usually not the case for experimental particles. Smoothing the particles corners can remove this difference. Furthermore, experimental data contains Poisson noise and thus empty pixels far from the central peak (see **d**) while our simulated data contains no noise and intensity visible everywhere, even far from the center.

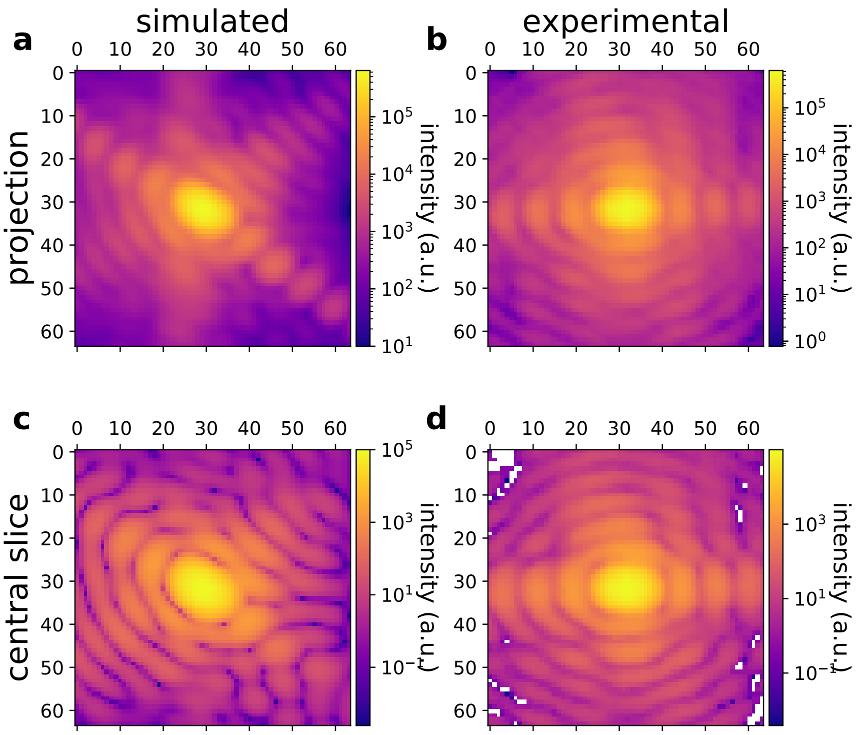

Fig. 2: Comparison of simulated and experimental BCDI data. a, **b** projection of simulated and experimental BCDI arrays. **c**, **d** corresponding central slice.

Additionally, the difference between simulated and experimental data can be visible in real space, when looking at the corresponding complex object $\mathbf{O}$. This object is calculated using the inverse Fourier transform (FT) of the complex BCDI amplitude $\mathbf{O} = \mathrm{FT}^{-1}[\mathbf{A}]$. The comparison of the object modulus $|\mathbf{O}|$ for simulated and reconstructed experimental data is shown in Supplementary Fig. 3. We can note that, the simulated object has sharper edges than the experimental one and some line artefacts are visible outside the object in Supplementary Fig. 3a. Furthermore, the histogram of $|\mathbf{O}|$ is shown in Supplementary Figs. 3c-d. The histogram's peak sharpness indicates the homogeneity of $|\mathbf{O}|$. Since the full width at half maximum (FWHM) is larger for the experimental peak (0.186) than for the simulated one (0.117), the simulated $|\mathbf{O}|$ is too homogeneous

compared to real experimental data.

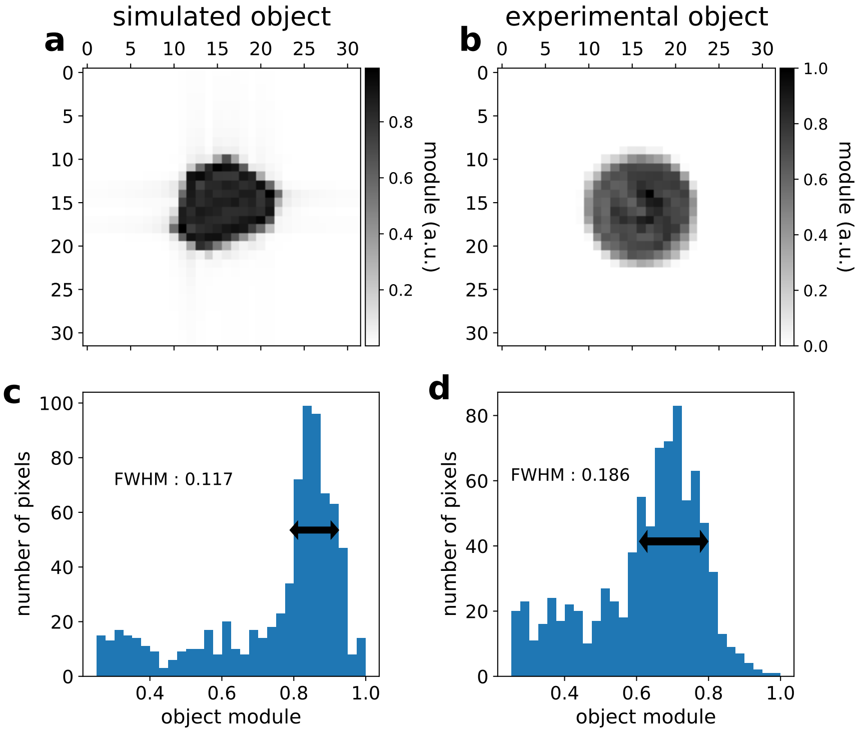

Fig. 3: Comparison between simulated and experimental reconstructed object. a, **b** are respectively the central slice of the simulated and experimental object modulus $|\mathbf{O}|$. **c**, **d** histogram of $|\mathbf{O}|$ showing that the peak of the experimental object is larger than the simulated one, meaning that the reconstructed experimental object module is less homogeneous than the simulated one.

S2.2 Real space modifications

In order to make our simulated data more similar to experimental ones, we first make modifications on the real space object $\mathbf{O}$ as shown in Supplementary Fig. 4. We use a gaussian convolution to smooth the particle boundaries and corners and remove all non-zero pixels outside a support (from **b** to **c**). Secondly, an additional random noise is added in order to remove the homogeneity of the object modulus ($|\mathbf{O}|$). This additional noise is a random Gaussian correlated profile as defined by *Wu et al.*[6], where the correlation length is randomly chosen for each simulated data.

Furthermore, the simulated object phase results from the particle relaxation using LAMMPS. In order to increase the dataset diversity and make our deep learning more robust, the phases of some simulated objects are replaced by a random Gaussian correlated profile with random phase

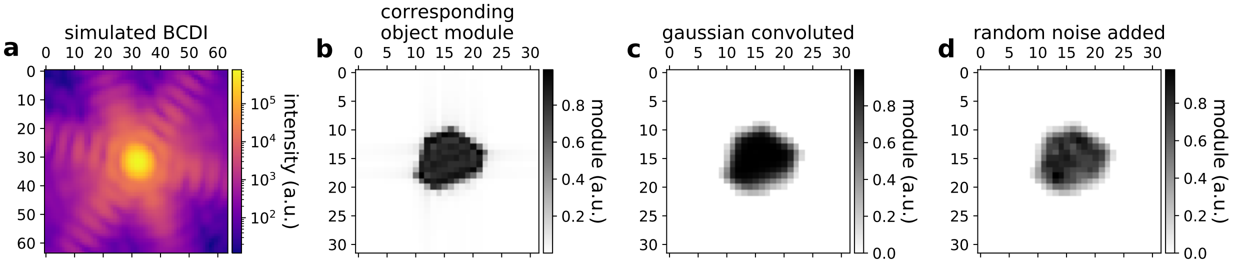

Fig. 4: Simulated object modulus modification. **a** Central slice of a simulated BCDI array. **b** Modulus of the corresponding complex object $|\mathbf{O}|$. **c** $|\mathbf{O}|$ after a real space Gaussian convolution. **d** $|\mathbf{O}|$ with an additional random Gaussian correlated noise.

amplitude. An example of a complete simulated object modification is shown in Supplementary Fig. 5.

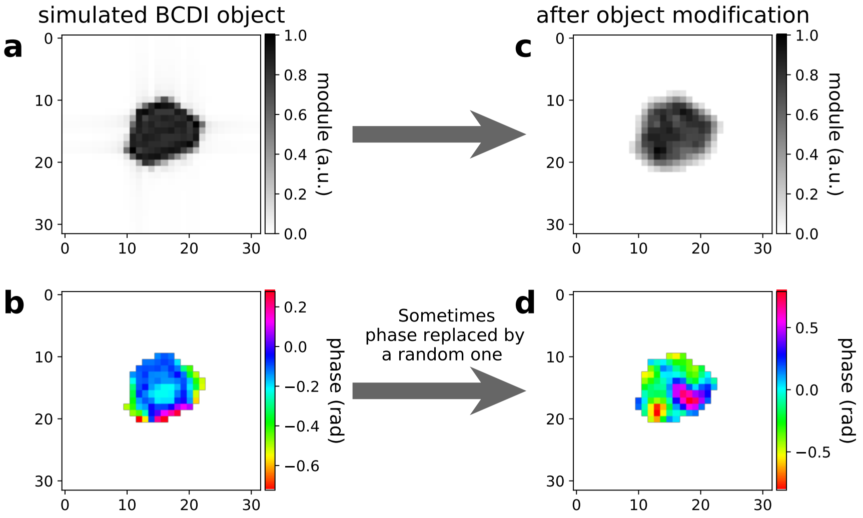

Fig. 5: Random simulated object modification. **a**, **b** Initial simulated BCDI object modulus and phase. **c** Modulus after Gaussian smoothing and random noise addition. **d** Some phases of simulated objects are replaced by a random Gaussian correlated profile with random amplitude.

S2.3 Reciprocal space modifications

Finally, after modifying the real space simulated object, we perform some additional corrections on the corresponding BCDI array $\mathbf{I} = |\mathrm{FT}[\mathbf{O}]|^2$. Supplementary Figs. 6 **a**-**b** show the original simulated BCDI array projection and central slice. The same ones are shown after the real space

object modification in Supplementary Figs. 6**c-d**. Our final adjustments shown in Supplementary Figs. 6**e-f** are a Gaussian convolution in order to smooth the Bragg peak fringes and Poisson noise with a random amplitude resulting in empty pixels far from the peak center (as it is the case on experimental data). These randomly modified simulated BCDI arrays are used to train our deep learning model and make it more robust for gap inpainting on experimental data.

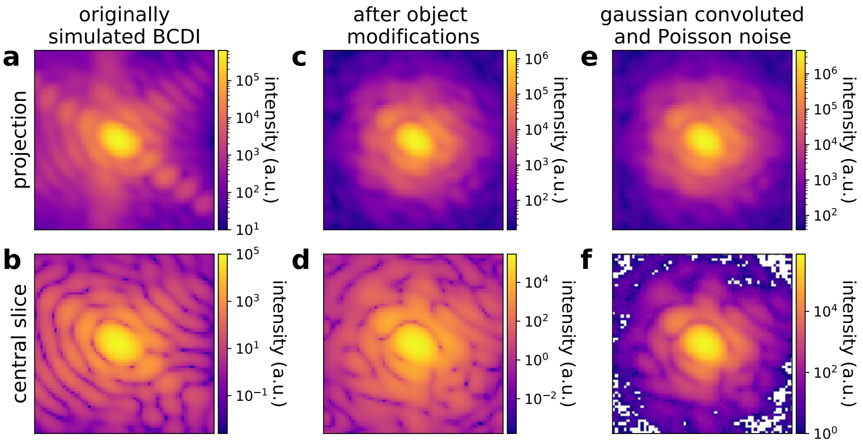

Fig. 6: Random modification of the simulated BCDI intensity. **a**, **b** Projection and central slice of the original simulated BCDI array. **c**, **d** Same after the real space object modifications. **e**, **f** Same after Gaussian convolution and applying Poisson noise statistic with random amplitude.

S3. SMALL PORTIONS PREDICTION

Our inpainting patching method presented in the main text allows for a smaller and more robust DL model able to yield accurate predictions on experimental data. The model is trained to make predictions on small 32x32x32 portions (**P**) of both simulated and experimental large BCDI data. Some examples of model prediction on small portions of simulated data are shown in Supplementary Fig. 7. The gap is randomly chosen to be either a fixed vertical gap in the middle of the array or a cross-shaped gap with a random vertical position (5th column in Supplementary Fig. 7 and 3rd-5th columns in Supplemenatry Fig. 8).

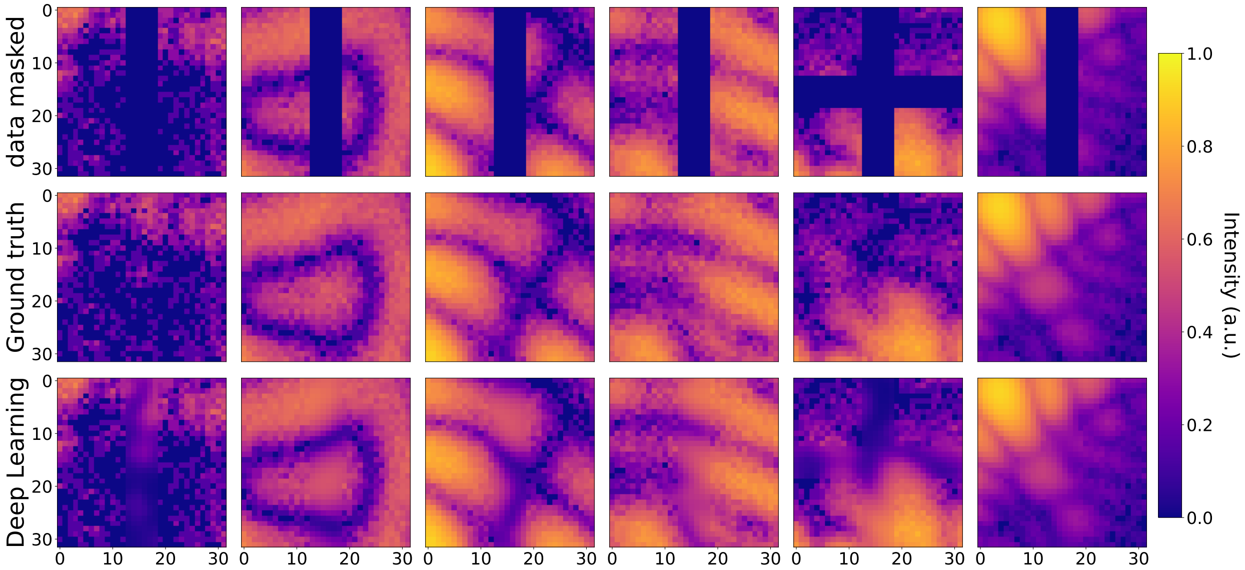

Fig. 7: Model prediction on small 32x32x32 pixel-size portions of simulated BCDI data. 1st row is the data masked with a random gap. 2nd row is the ground truth and 3rd row is the deep learning model prediction.

Our model predictions on small portions of experimental data acquired at the ID01 beamline of the ESRF-EBS are shown in Supplementary Fig. 8. The results are similar to the ones obtained with simulated data, showing that the use of experimental data during training makes the model robust. This eventually allows for efficient predictions on large experimental BCDI data with gap.

One can note that our model prediction is “smoother" in low intensity regions compared to the ground truth (see 2nd and 4th columns in Supplementary Fig. 7). This is explained by the fact that the “grainy" aspect of the ground truth in low intensity regions is due to Poisson noise. Since this noise is random, the model can not predict the specific noise configuration inside the gap and

thus the best prediction the model can make is the noise average. This phenomenon was already reported in the literature like in the Noise2Void denoising method[7].

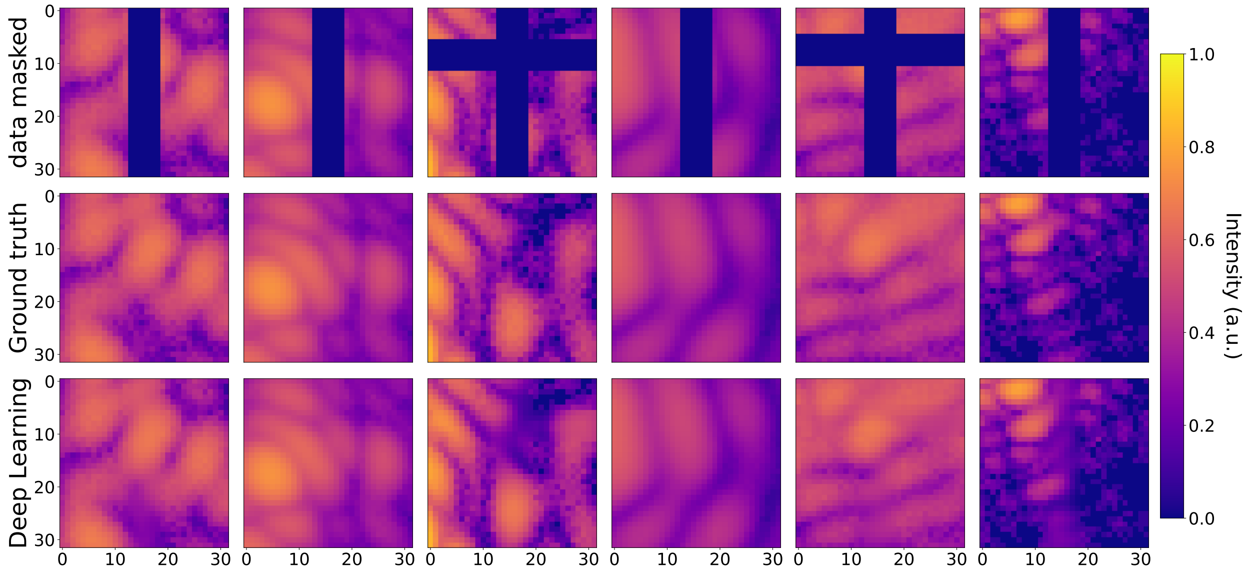

Fig. 8: Model prediction on small 32x32x32 pixel-size portions of experimental BCDI data. 1st row is the data masked with a random gap. 2nd row is the ground truth and 3rd row is the deep learning model prediction.

S4. COMPARISON WITH STANDARD INTERPOLATION INPAINTING

In this section, for the sake of completeness, we show the in-gap predictions provided by nearest-neighbor and cubic interpolations.

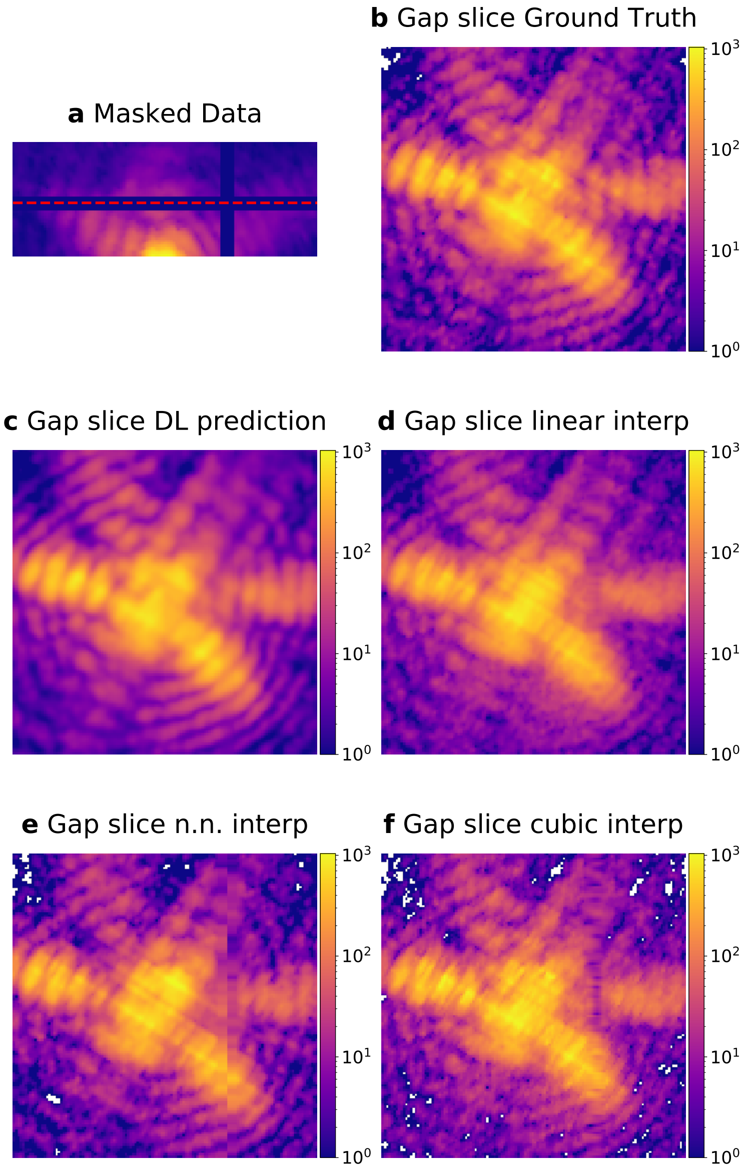

Fig. 9: In-gap slice comparison between DL prediction and standard interpolation on experimental BCDI data. **a** Original data masked with a cross-like 6 pixel-wide gap. **b** Ground truth in-gap slice of the non-masked data along the red dotted line in **a**. **c** In-gap slice prediction using DL model, **d** using linear interpolation, **e** nearest - neighbor interpolation and **f** cubic interpolation.

S5. SKIP PIXEL FOR LARGE PREDICTION

Our patching method consists in sliding a 32x32x32 pixel-size portion (**P**) over the gap area and making a prediction at each position. These predictions are then averaged in overlapping regions. This technique also makes our model more robust when predicting experimental data since potential artefacts are averaged out. This sliding technique has 1 free parameter: the number of “skipped pixels" k which represents by how much **P** is shifted between one small prediction and the next one along the gap. One can choose to shift **P** by only one pixel at a time ($k = 0$), obtaining the best prediction possible. However, this takes up to 11 minutes for a 128x128x128 pixel-size BCDI data array with a cross-shaped gap. Moving **P** by more than 1 pixel at a time, the prediction time rapidly decreases following a power law as shown in Supplementary Fig. 10.

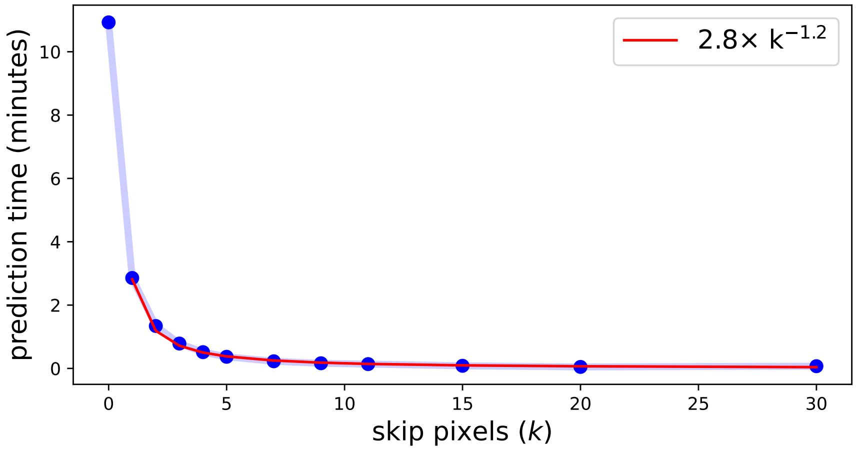

Fig. 10: Prediction time for a 128x128x128 pixel-size BCDI array with a cross-shaped gap as a function of the skip pixels (k). The computation time decreases as k increases.

Therefore, skipping pixels can be a solution to make a fast prediction on a large BCDI array. However, skipping too many pixels also worsens the prediction accuracy. In Supplementary Fig. 11**a**, we show the ground truth detector slice in the middle of the gap. The prediction is then performed on the masked data with different skip pixel values and the results are shown in Supplementary Figs. 11**b-d**. Using a skip pixel value of 4 (Supplementary Fig. 11**c**) leads to a drastic decrease of the prediction time (from 11m56s down to 1m31s, a factor ∼8) without visible modifications. However, the drawback of using a large skip pixel value of 30 (Supplementary Fig. 11**d**) is the occurrence of square shaped artefacts in the prediction.

The averaged error as a function of the skip pixel value is shown in Supplementary Fig. 12.

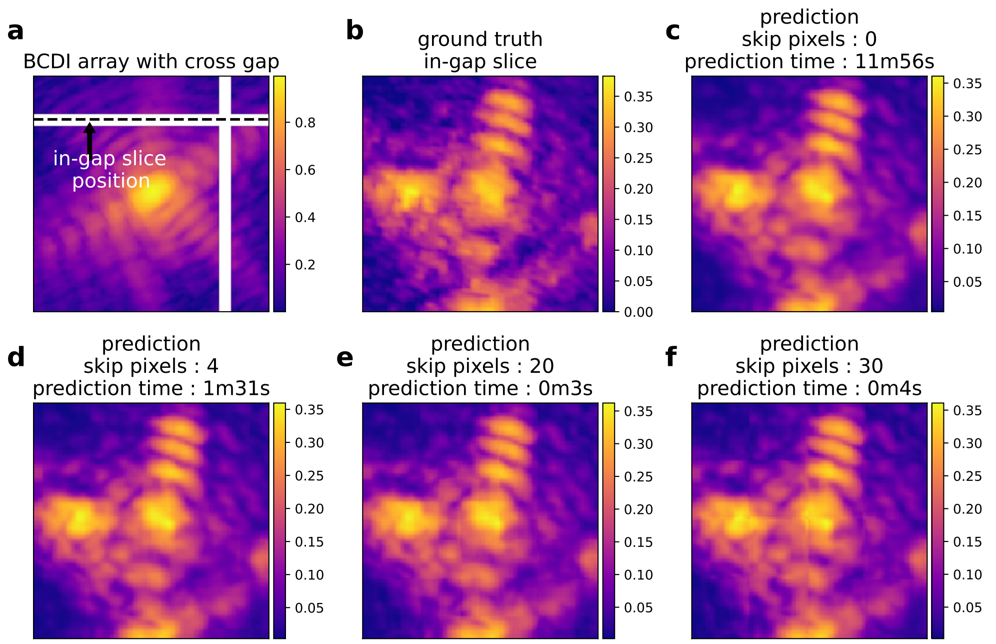

Fig. 11: Predicted in-gap slice for different skip pixels. a BCDI array with a cross shaped gap shown in white. The in-gap slice position is shown by a black dotted line. **b** Ground truth in-gap slice of experimental BCDI data. **c** Deep learning prediction without skipped pixels (k=0). **d** Prediction with k = 4. **e** Prediction with k = 20. **f** Prediction with k = 30. In that case, square prediction artefacts are now visible.

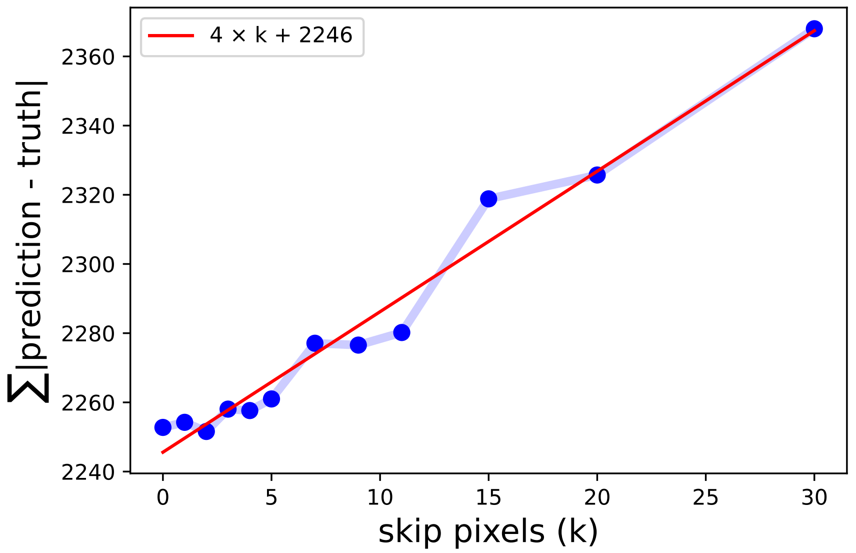

Fig. 12: Trend of the sum of the absolute errors between the DL prediction and the ground truth as a function of the skip pixel.

S6. ACCURACY *VS* OVERSAMPLING

In order to calculate the accuracy as a function of oversampling shown in Figure 6 of the main text, we simulated the same BCDI array region for several oversampling conditions. The model prediction was then computed for the whole image following the procedure illustrated in Supplementary Fig. 13. The model prediction is computed for different gap positions (Supplementary Figs. 13**a**-**b**-**c**). These predicted gaps (**d**-**e**-**f**) are then combined to produce the full predicted image (**g**). The accuracy, expressed in terms of the Pearson Correlation Coefficient is finally calculated, comparing the predicted image with the ground truth.

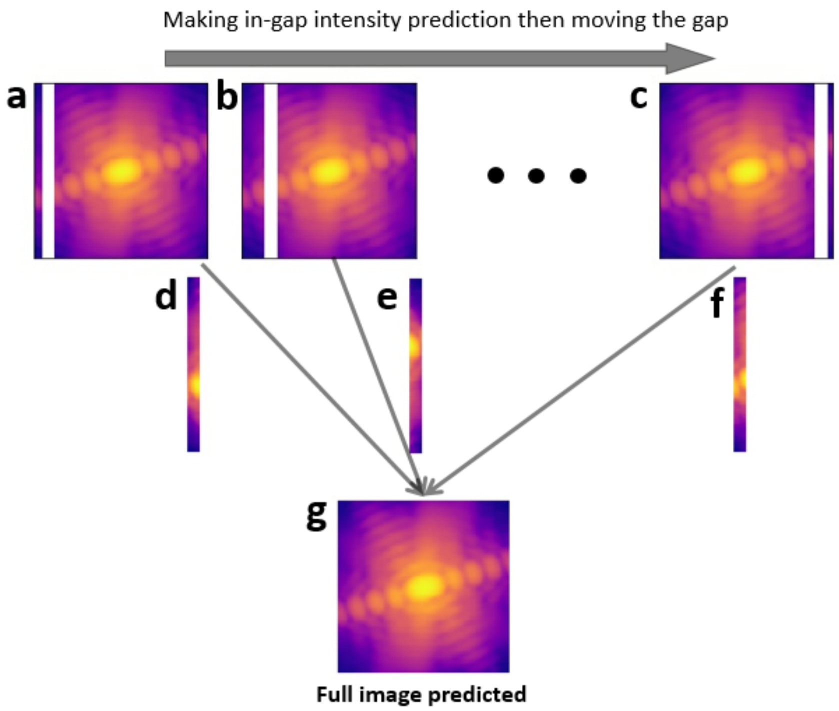

Fig. 13: Schematic of the accuracy *vs* oversampling calculation. a-**b**-**c** Ground truth BCDI array on which a gap is applied in different positions and then predicted using our DL model. **d**-**e**-**f** Predicted gap regions. **g** Full predicted image made by combining all the predicted gaps.

Supplementary Fig. 14 shows an example of full BCDI array predictions at different oversampling ratios and for models with different gap sizes. As expected, the prediction gets worse as the oversampling becomes small and as the gap size becomes larger.

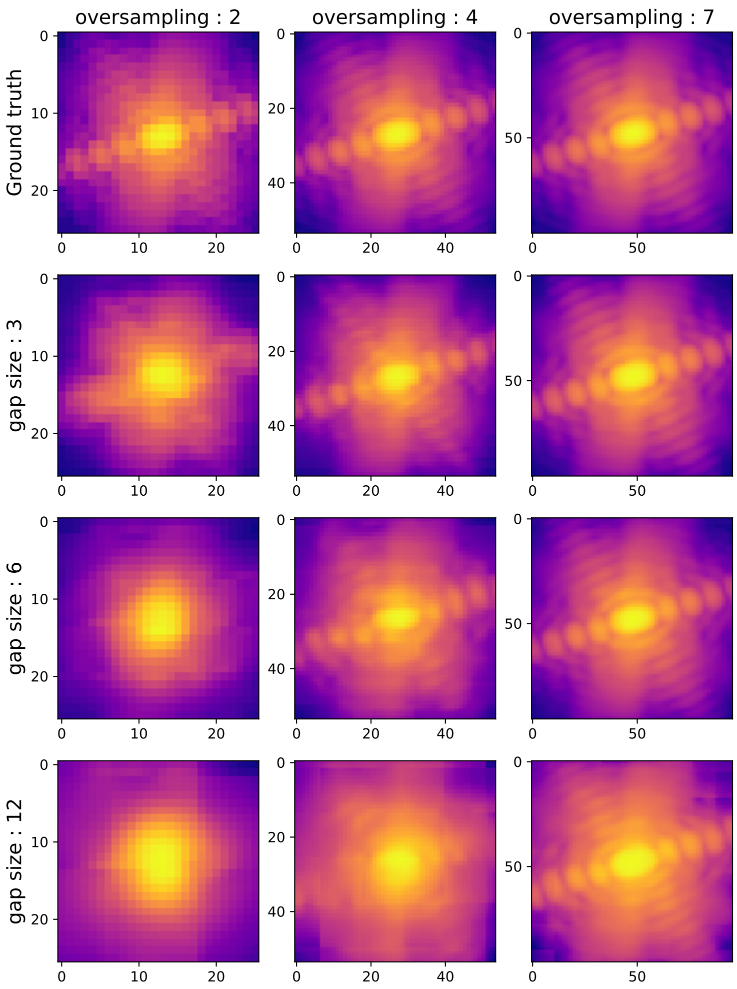

Fig. 14: Full image prediction at different oversampling values and gap sizes. 1st row is the ground truth BCDI arrays at different oversampling values. 2nd-3rd-4th rows show the full images predicted using DL models at different gap sizes. Best predictions occur for large oversampling and small gap size.

S7. REAL-SPACE RESULTS

In this section, we provide further details about the inpainting results shown in Figure 7 of the main text. The central slices of the corresponding BCDI intensities are shown in Supplementary Fig. 15, respectively for the unmasked data (**a**), masked (**b**) and DL inpainted (**c**).

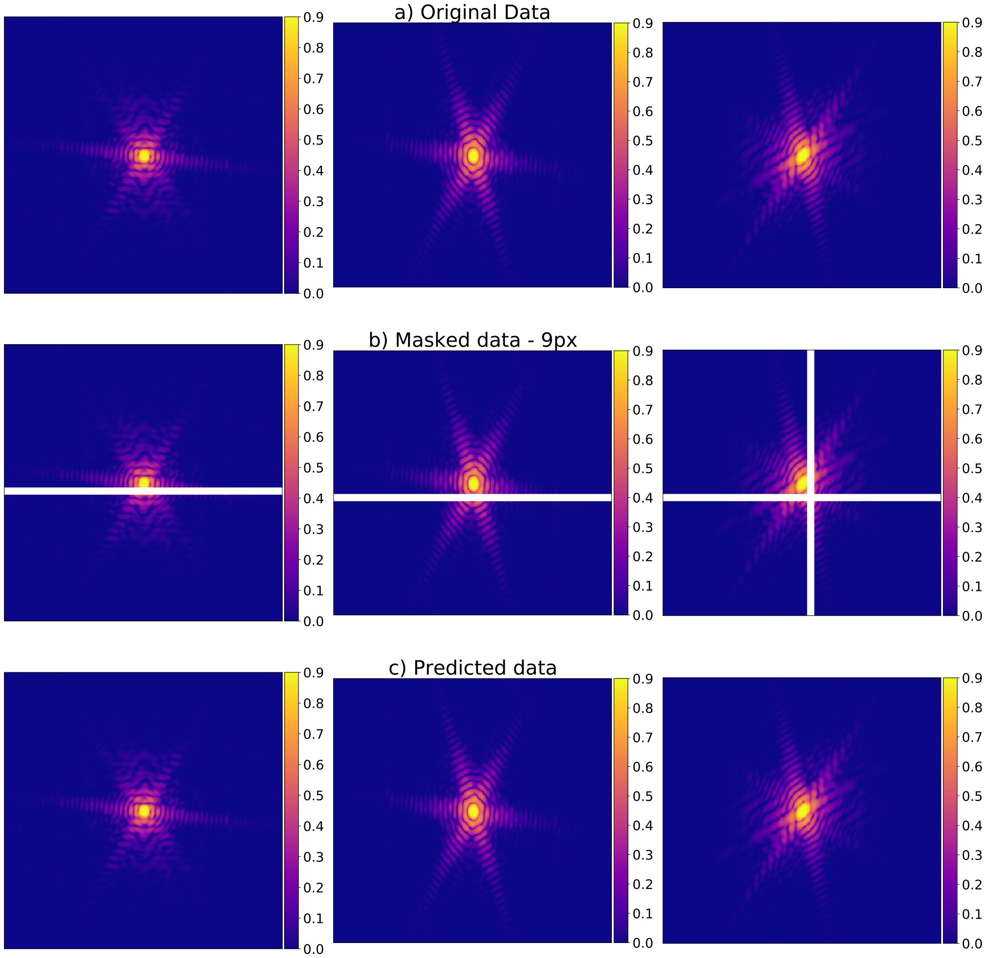

Fig. 15: Central slices along the three orthogonal planes of the simulated diffraction pattern treated in Ref.[8] for the **a** Ground truth case, **b** masked with 9 pixel-wide cross-shaped gap case and **c** DL inpainted case.

Moreover we present in Supplementary Fig. 16 the corresponding object phase for different gap widths in the simulated diffractions. As explained in the main text, the simulated phase was set to zero in the ground truth to facilitate straightforward comparisons. The particle phase variations increase with the gap size (2nd row of Supplementary Fig. 16), while the DL inpainting phase (3rd row) remains low even with the large 12 pixel-wide gap.

From the reconstructed objects at different gap widths of Supplementary Fig. 16, we calculated

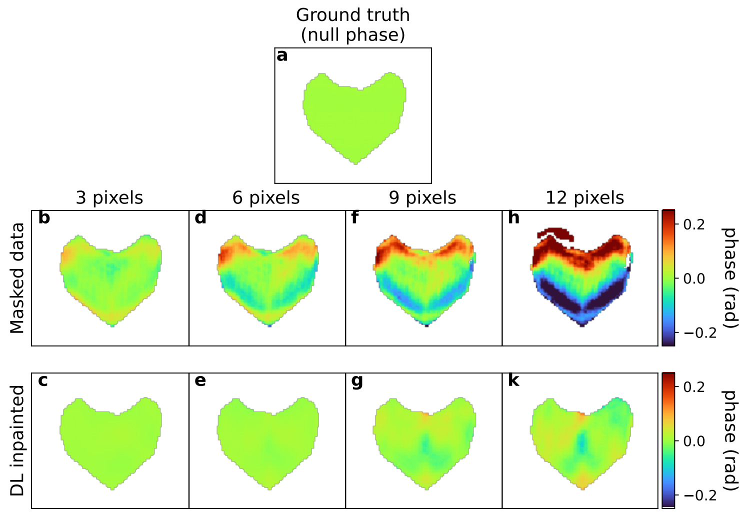

Fig. 16: Object phase comparison for different gap sizes. a Ground truth null phase. **b-d-f-h** Phase for masked data with different gap sizes. **c-e-g-k** Corresponding phase after DL inpainting.

the average strain per gap width shown in Supplementary Fig. 17. This average strain is dropping for the reconstructed object with gap (in blue) since it introduces an asymmetry in the BCDI peak. However, our DL inpainting (in red) corrects this artefact and the average strain stays very close to zero value as it is the case for the ground truth. Variations from the average strain are represented as error bars in Supplementary Fig. 17. It shows that the strain heterogeneity (visible in Supplementary Fig. 16) is larger for the case of the masked diffraction with respect to the DL inpainted one.

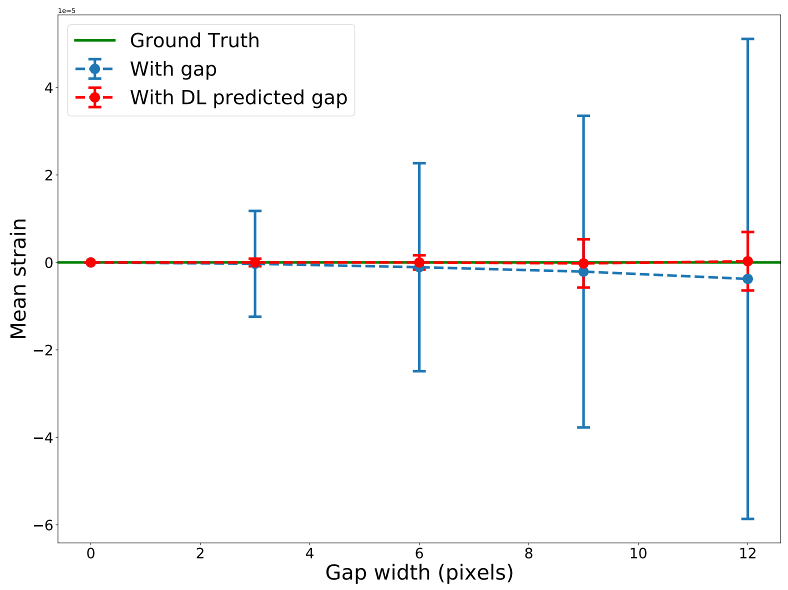

Fig. 17: Average strain. Calculation for different gap sizes for both masked and DL inpainted diffraction patterns.

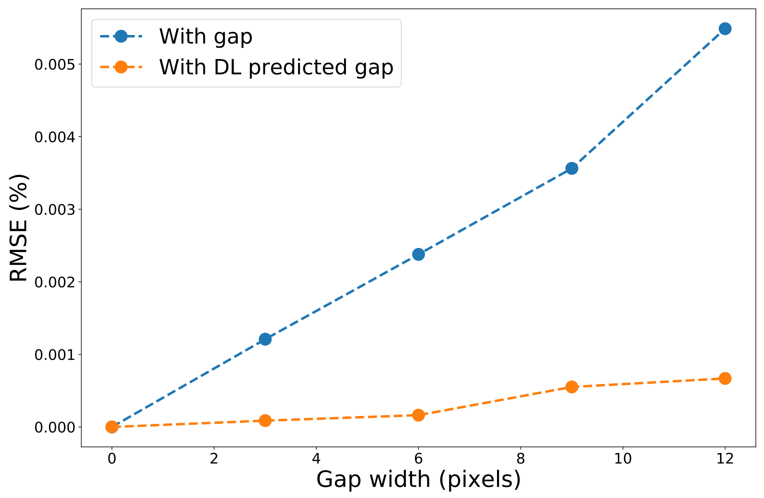

Fig. 18: Calculation of the zero-average strain root mean squared error (RMSE) vs the gap size for both cases of masked and DL inpainted diffraction patterns. For all gap sizes, the DL inpainted diffraction patterns yield a smaller error.

S6. INPAINTING AS A SOLUTION FOR HIGH RESOLUTION DATA

In case of large BCDI arrays used in high-resolution reconstructions, artefacts from the detector gaps can be problematic. A typical example is shown in Supplementary Fig. 1**a** where the detector is relatively far from the center of the Bragg peak. However, given that the data size is 256×300×300 pixel-size, the number of in-gap pixels is large enough to create artefacts on the reconstructed object.

The reconstructed intensity (square modulus of the Fourier transform of the reconstructed object) is shown in Supplementary Fig. 19 for several preprocessing methods. Supplementary Fig. 19**a** illustrates the reconstructed intensity where a mask is used during the iterative reconstruction, leaving pixels free in the gap. However, this technique often leads to an abnormally high reconstructed in-gap intensity. In **b**, the same mask method is used but the mask is only applied near the peak streaks. In **c**, the gap is filled with 0's. Finally, in **d**, the gap is inpainted using our Deep Learning method. A zoomed-in version is shown in Supplementary Fig. 23.

Supplementary Fig. 20 shows the corresponding reconstructed object modulus for the different methods. Oscillatory artefacts are clearly visible in **a** and **b** where a mask of free pixel was used during the iterative reconstruction. This is due to the additional in-gap reconstructed intensity observed for these 2 methods. When leaving the gap filled with 0's, the oscillations are less important as seen in **c** but are still visible. On the other hand, the reconstruction using Deep Learning inpainting shown in **d** does not show this artefact anymore. We emphasize that Deep Learning inpainting does not remove any high frequency information from the reconstruction, leading to the most reliable object reconstruction in this case.

Supplementary Fig. 21 shows the reconstructed strain for the different methods. Since the strain is calculated from the phase gradient, the oscillatory artefact becomes even more important. As for the object modulus, the Deep Learning method (**e**) shows less oscillatory artefacts compared to the other methods in Supplementary Fig. 21**a**-**b**-**c**.

Supplementary Fig. 22 shows a second example of large BCDI array DL inpainting on a particle containing a dislocation. In this case as well, small oscillation artefacts on the strain maps are visible when in-gap pixels are left free during phase retrieval (**b**-**c**). Our DL inpainting manages to remove these artefacts (**e**-**f**).

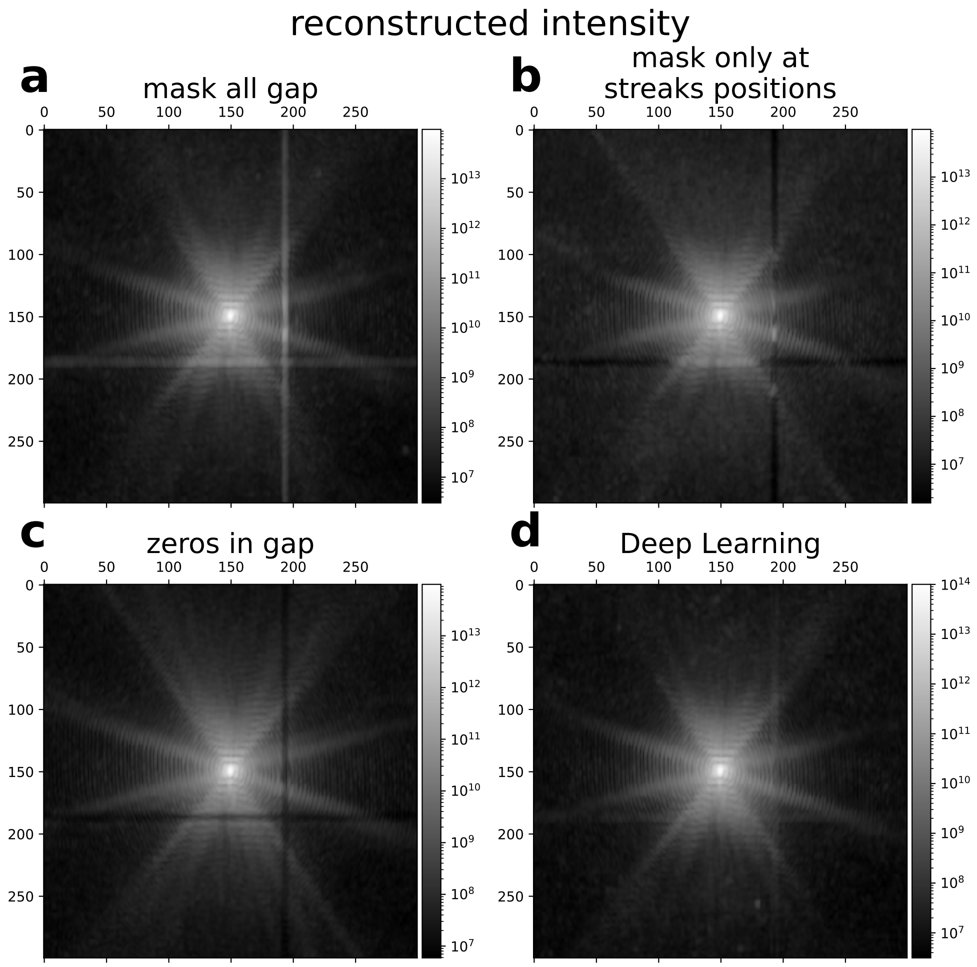

Fig. 19: Reconstructed BCDI intensity comparison with different methods on experimental large BCDI array. (in gray scale for the artefacts visibility) **a** in-gap pixels free during the reconstruction. **b** free only in-gap pixels close to the BCDI streaks. **c** Leave 0's inside the gap. **d** Deep-Learning inpainting.

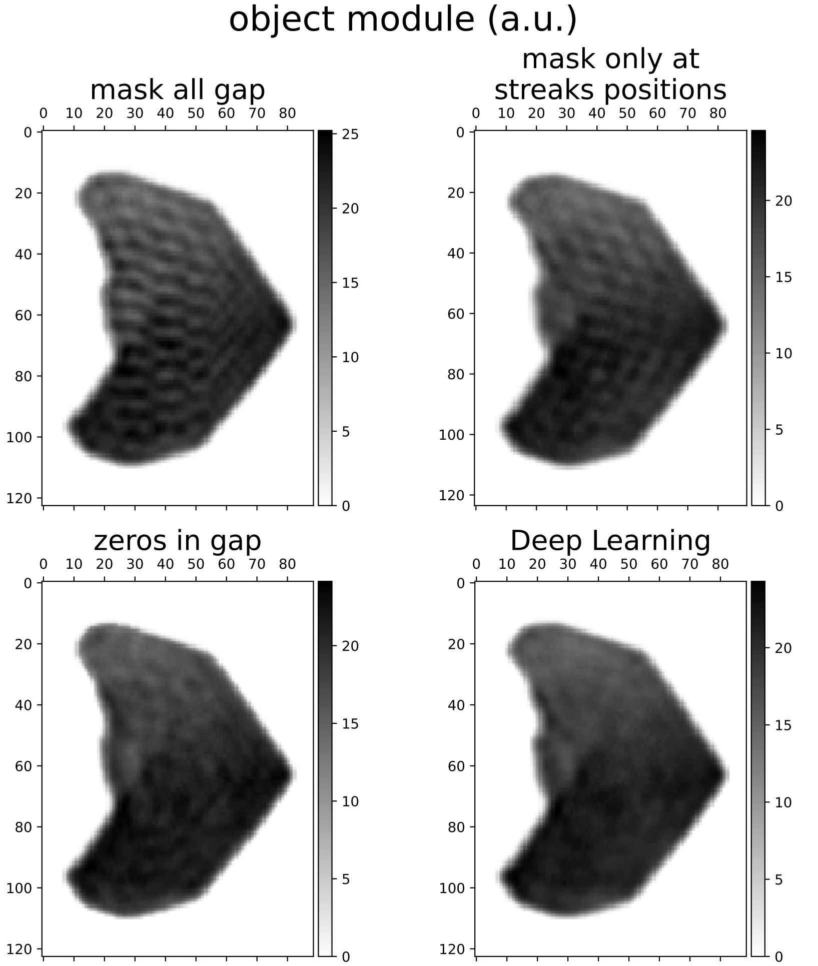

Fig. 20: Reconstructed object modulus comparison with different methods on experimental large BCDI array. Oscillatory artefacts are observed (having different magnitudes) for all methods except the Deep-Learning inpainting.

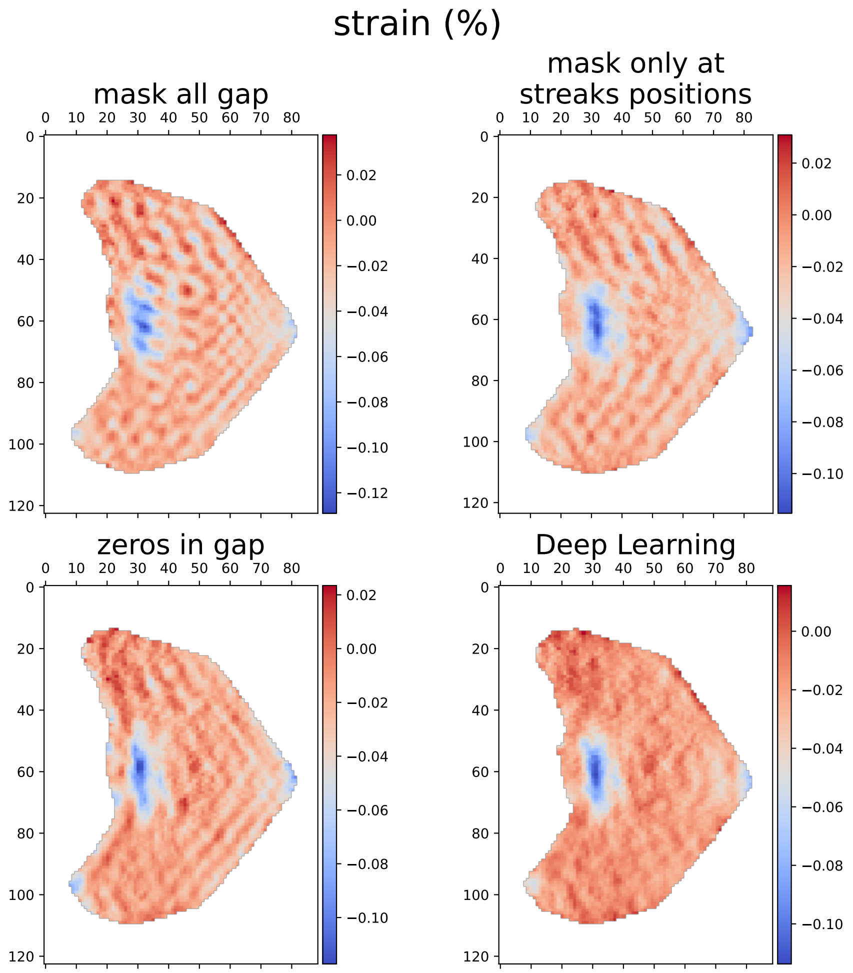

Fig. 21: Reconstructed object strain comparison with different methods on experimental large BCDI array. Oscillation artefacts are even more problematic on the strain map. Again, the Deep-Learning inpainting removes most of the oscillations.

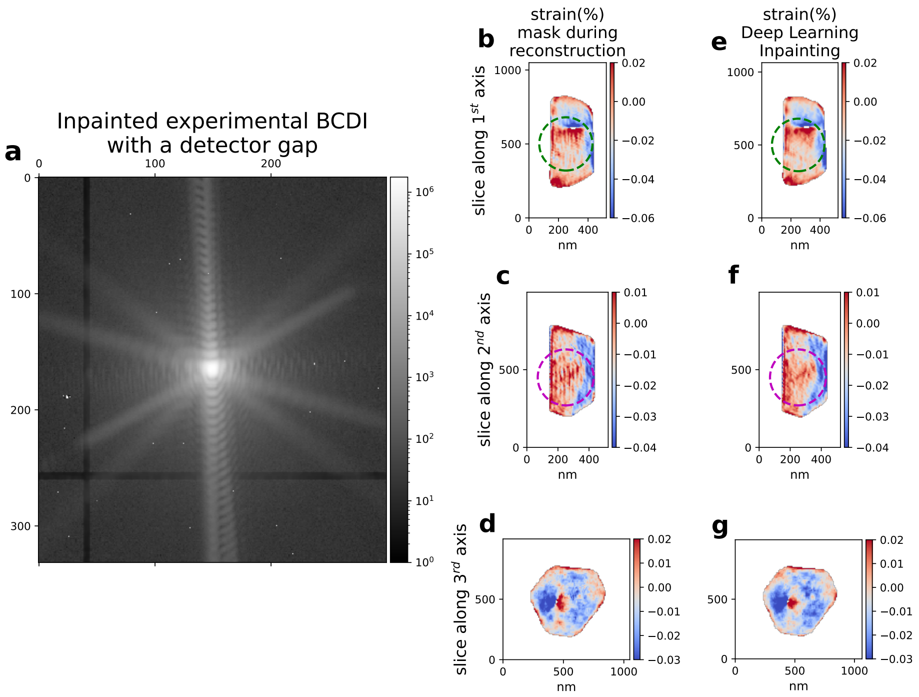

Fig. 22: 2nd example of experimental large BCDI data inpainting. a Projection of the inpainted BCDI array. **b**-**c**-**d** Strain reconstruction with free in-gap pixels. **e**-**f**-**g** Strain after DL inpainting. Broken lines circles indicates region were the oscillation artefacts disappeared after DL inpainting.

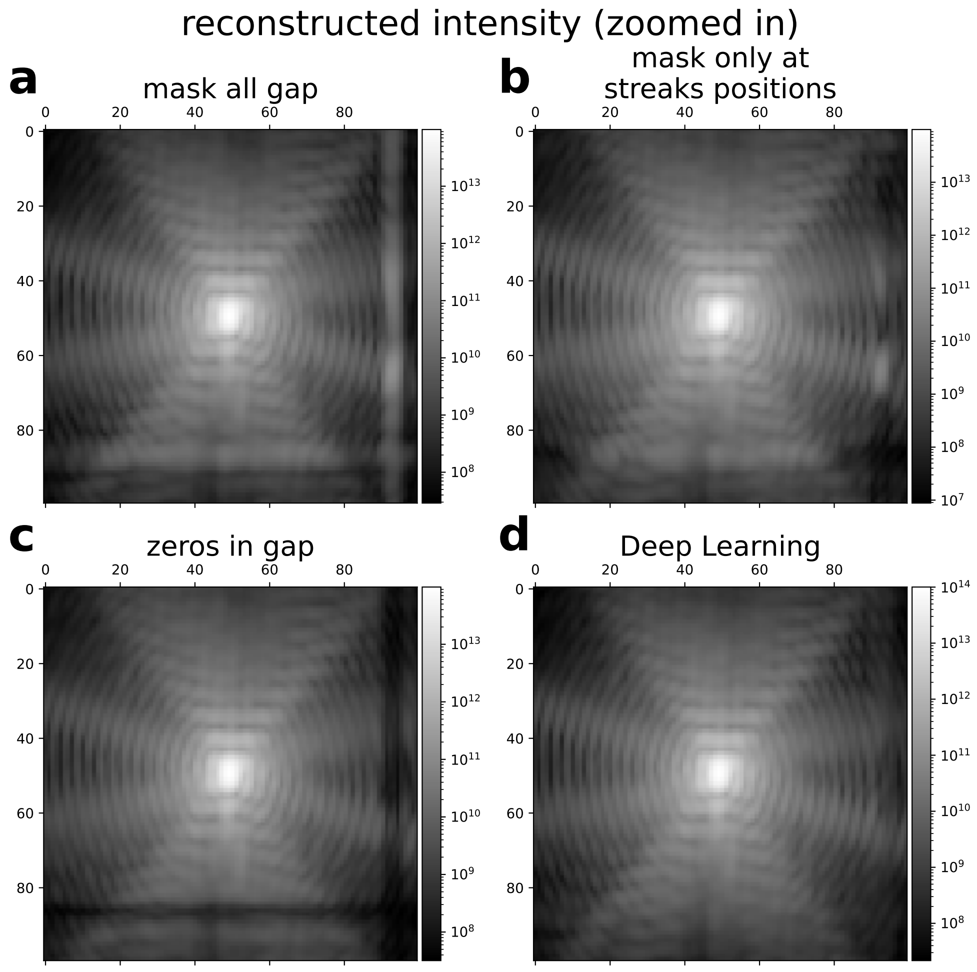

Fig. 23: zoomed-in version of Supplementary Fig. 19 around the central Bragg peak.

S7. GAP RECONSTRUCTION USING UNSUPERVISED LEARNING

When leaving the gap pixels free during the phase retrieval process, standard iterative algorithms tend to create abnormally high intensity in the gap as shown in Supplementary Fig.19**a**. Several papers proposed to use machine learning (ML) unsupervised reconstruction as a new method for phase retrieval[6,9]. In order to test the gap effect using ML methods, we created a real object (with a null phase) shown in Supplementary Fig.24**a**. The corresponding BCDI array (absolute squared Fourier Transform of the object) is shown in Supplementary Fig.25**a**. We then simulated a detector gap by removing pixels' intensity as illustrated in Sup. Fig.25**b**.

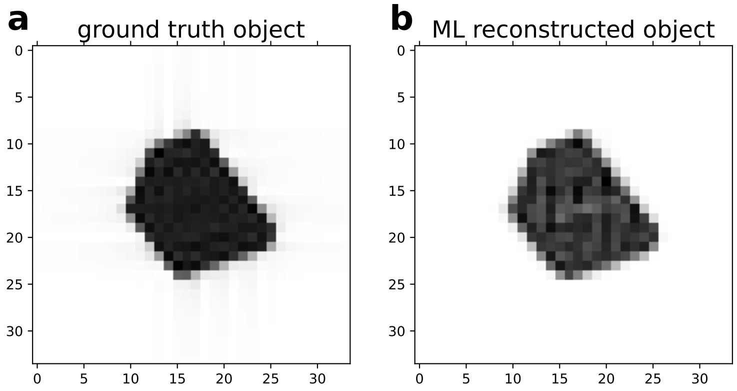

Fig. 24: a Ground truth object (with null phase). **b** Reconstructed object using unsupervised Machine Learning.

In order to make ML unsupervised phase retrieval on the gapped BCDI, we used the model architecture proposed in[9]. We left the gap pixels out of the loss function, free to take any values as long as the object support constraint is satisfied. The reconstructed object is shown in Sup. Fig.24**b**. The corresponding reconstructed intensity is shown in Sup. Fig.25**c**. We don't observe an abnormally high reconstructed intensity in the gap compared to standard iterative algorithms, indicating the unsupervised ML might be more robust when a gap is present in the measured BCDI data.

However, there are a few drawbacks to the unsupervised ML method presented here. (1) In our case, we have a very simple small simulated object (null phase). We don't expect unsupervised ML to work as good for strained experimentally measured BCDI data. (2) This fully unsupervised ML phase retrieval takes a few minutes for 1 reconstructions while iterative phase retrieval are much faster. One could use a pre-trained model in order to accelerate the ML model training but

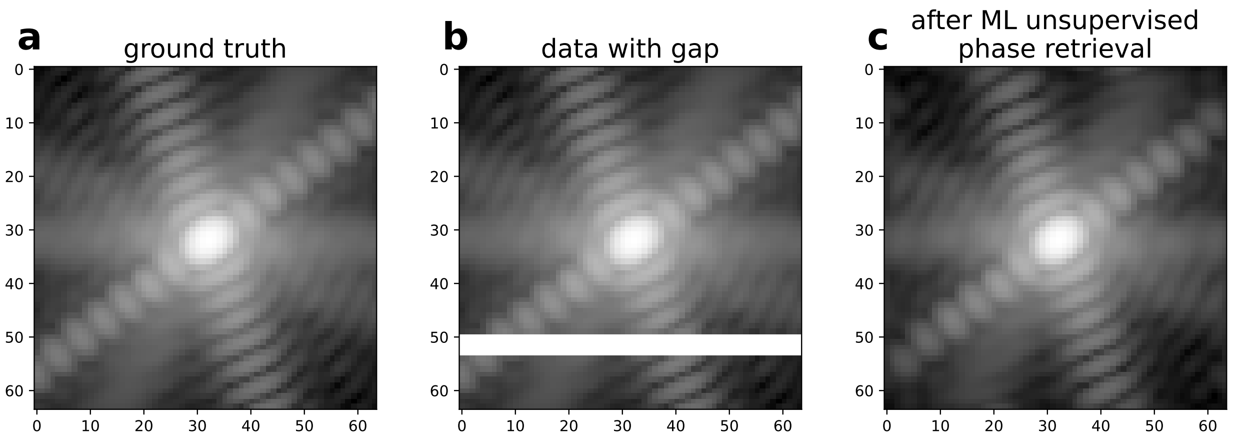

Fig. 25: a b c

that would require a large training dataset. Furthermore, the presented BCDI array in Sup. Fig.25 is rather small (64x64x64). ML phase retrieval on a large BCDI array would require more memory and would take longer to train. For these reasons, we don't expect unsupervised ML to replace standard iterative algorithms yet, thus the need for our gap inpainting method on experimental BCDI data.

REFERENCES

[1]Bruce Lim, Ewen Bellec, Maxime Dupraz, Steven Leake, Andrea Resta, Alessandro Coati, Michael Sprung, Ehud Almog, Eugen Rabkin, Tobias Schulli, and Marie Ingrid Richard. A convolutional neural network for defect classification in Bragg coherent X-ray diffraction. *npj Computational Materials*, 7(1), 12 2021.

[2]David Rodney. Merlin in a nutshell. Unpublished, 2010.

[3]Steve Plimpton. Fast parallel algorithms for short-range molecular dynamics. *Journal of Computational Physics*, 117(1):1–19, 1995.

[4]Vincent Favre-Nicolin, Gaétan Girard, Steven Leake, Jerome Carnis, Yuriy Chushkin, Jerome Kieffer, Pierre Paleo, and Marie Ingrid Richard. PyNX: High-performance computing toolkit for coherent X-ray imaging based on operators. *Journal of Applied Crystallography*, 53:1404–1413, 10 2020.

[5]The oversampling ratio is defined for each dimension as the ratio between the array size and the crystal size in direct space. According to Nyquist theorem, to reconstruct the signal in direct space it is necessary to sample at a frequency at least double the maximum frequency of the signal. In an experiment this means moving the detector along the propagation direction until the resolution on the detector is at least of two pixels per fringe.

[6]Longlong Wu, Shinjae Yoo, Ana F. Suzana, Tadesse A. Assefa, Jiecheng Diao, Ross J. Harder, Wonsuk Cha, and Ian K. Robinson. Three-dimensional coherent X-ray diffraction imaging via deep convolutional neural networks. *npj Computational Materials*, 7(1), 12 2021.

[7]Alexander Krull, Tim-Oliver Buchholz, and Florian Jug. Noise2void-learning denoising from single noisy images. In *Proceedings of the IEEE/CVF conference on computer vision and pattern recognition*, pages 2129–2137, 2019.

[8]Jérôme Carnis, Lu Gao, Stéphane Labat, Young Yong Kim, Jan P. Hofmann, Steven J. Leake, Tobias U. Schülli, Emiel J.M. Hensen, Olivier Thomas, and Marie Ingrid Richard. Towards a quantitative determination of strain in Bragg Coherent X-ray Diffraction Imaging: artefacts and sign convention in reconstructions. *Scientific Reports*, 9(1), 12 2019.

[9]Yudong Yao, Henry Chan, Subramanian Sankaranarayanan, Prasanna Balaprakash, Ross J. Harder, and Mathew J. Cherukara. AutoPhaseNN: unsupervised physics-aware deep learning of 3D nanoscale Bragg coherent diffraction imaging. *npj Computational Materials*, 8(1), 12 2022.
